# Supplementary material for: Combined Lifestyle Interventions in the Prevention and Management of Asthma and COPD: A Systematic Review
Source: Nutrients. 2024 May 17;16(10):1515. doi: 10.3390/nu16101515 (PMC11124109; doi:10.3390/nu16101515)
Supplement: Supplementary file 1 [file nutrients-16-01515-s001.zip › nutrients-2985894-supplementary.pdf]

Appendix

Methods

Data source and search strategy

A systematic literature search was conducted in MEDLINE via PubMed, EMBASE via OVID, and PsycInfo. The initial search was performed on 22 June 2022 and repeated on 24 August 2023 to check whether new articles had been published in the past year. The search strategy included relevant terms for the population, intervention and study design (PICOS) (Table A1). The search strategy was not restricted on comparison since all comparators were eligible for inclusion. No search terms on outcomes were included due to large heterogeneity in the relevant behavioural and physiological outcomes. Using additional search terms, the search was limited to intervention studies (i.e. single group pre-post studies, randomised controlled trials (RCTs) and non-randomised controlled trial studies). Boolean operators were used to narrow down the search and Medical Subject Headings (MeSH) were used to make sure all relevant articles were found. In order to get a complete overview of the available literature, no publication date limit was implemented. Reference lists of relevant studies were also screened for additional studies that were not identified with the initial search.

Study selection and eligibility criteria

Articles obtained with the search were first screened based on title and abstract for relevance, after which relevant full-text articles were assessed for eligibility. Two researchers (C.D.C. Born and R. Bhadra) independently performed the screening of the obtained articles in Rayyan [1]. When uncertainties or disagreements arose, they were resolved through discussion and – if needed – by consulting a third investigator (R.J.H.C.G. Beijers). Studies that targeted the prevention and management of asthma or COPD, and as such included patients diagnosed with asthma or COPD or individuals at high risk of developing asthma or COPD as defined by the authors (>18 years old), were eligible for inclusion (Table A1). Additionally, articles were considered for inclusion when they reported on CLIs that targeted a behavioural change in at least two of the following lifestyle factors: PA levels, dietary intake, smoking behaviour, alcohol consumption, stress levels, or sleeping behaviour. Moreover, in case of RCTs and non-randomised controlled trial studies, the intervention group had to differ from the control group in at least two lifestyle factors being targeted. Studies focussing on environmental

changes to modify behaviour (e.g., constructing more bicycle lanes to stimulate PA, increasing availability of fruits and vegetables at local supermarkets, limiting sales points for tobacco) were excluded. Furthermore, studies including CLIs in combination with pharmacological treatment were excluded since the effects of lifestyle interventions were the primary focus of the review. Studies primarily focussing on the effects of nutritional supplementation or physical exercise training were also excluded, because nutritional supplementation and exercise training alone were not considered CLI components. The CLIs needed to be delivered human-to-human, via physical or digital contact, and could be implemented either as individual or group interventions. Studies conducted in an inpatient setting were excluded, because the review interest was in CLIs that were conducted in a community (-dwelling) setting. Only publications in the English language and research articles for which results were available were included. Single group pre-post studies, RCTs and non-randomised controlled trial studies were eligible for inclusion. Lastly, studies were only included if they reported PA levels, dietary intake, smoking behaviour, alcohol consumption, stress levels, sleeping behaviour, QoL, or physiological outcomes. In case of RCTs and non-randomised controlled trial studies, between group differences needed to be reported.

Data extraction and synthesis

Data extraction was performed by two reviewers (C.D.C. Born and R. Bhadra), who crosschecked each other's extraction (data extraction form available on OSF). Extracted data included publication characteristics, study design, participant baseline characteristics (mean age and Body Mass Index (BMI), sex, smoking status, Forced Expiratory Volume in one second (FEV1), comorbidities), details of the CLIs (characteristics/components) and comparator, outcomes, and moments in time when outcomes were measured. Regarding the outcomes, data on the effects of the CLIs on PA levels, dietary intake, smoking behaviour, alcohol consumption, stress levels, and sleeping behaviour were extracted. Additionally, data on the effects of the CLIs on other outcomes, including quality of life and physiological outcomes (i.e. weight, lung function, respiratory symptoms, exercise capacity, and muscle strength) were extracted. The data were analysed separately based on the reported CRD. Effects of the CLIs on the before mentioned outcomes were presented in a table using colour coding. The colour green was used to signify a significant desired effect on the outcome, grey to signify no change in outcome (non-significant effects), and red to signify a significant undesired effect on the

outcome. Desired effects were interpreted as improvements in the reported outcomes from a health perspective, which was sometimes dependent on the study population and aim of the specific study (e.g., weight gain could be desired in underweight patients while weight loss could be desired in overweight patients). Regarding the time points for which the outcomes were reported; the first measurement after the end of the intervention was reported, as well as all additional follow-up measurements. For RCTs and non-randomised controlled clinical trials, between group effects were reported and for single group pre-post studies within group differences were reported.

Risk of bias (quality) assessment

Quality of the included studies was assessed using the Quality Assessment Tool for Quantitative Studies, developed by the Effective Public Health Practice Project at McMaster University [2]. This tool is used to evaluate the quality (indicated as "strong," "moderate", or "weak") of selected studies in each of the following eight domains: (1) selection bias, (2) study design, (3) confounders, (4) blinding, (5) data collection methods, (6) withdrawals and dropouts, (7) intervention integrity, and (8) analyses. The score in each of the areas was combined to compute an overall quality score for every study included in the review. An article received the overall score "strong" in case of no weak ratings, "moderate" in case of one weak rating and "weak" in case of two or more weak ratings in any of the aforementioned domains. The quality check was performed by two reviewers (C.D.C. Born and R. Bhadra), who crosschecked each other's quality checks. When uncertainties or disagreements arose, they were resolved through discussion and – if needed – by consulting a third investigator (R.J.H.C.G. Beijers).

Table S1 PICOS criteria for inclusion of studies

**Parameter**	**Inclusion criteria**
Population	Patients diagnosed with asthma or chronic obstructive pulmonary disease or individuals defined as being at high risk of developing asthma/chronic obstructive pulmonary disease (>18 years old).
Intervention	Combined lifestyle interventions that target a behavioural change in at least two of the following lifestyle factors:<br>- Physical activity levels<br>- Dietary intake<br>- Smoking behaviour<br>- Alcohol consumption<br>- Stress levels<br>- Sleeping behaviour<br>The delivery mode is human-to-human via physical or digital contact, implemented either as individual or group interventions.
Comparison	All comparators are eligible for inclusion (e.g. true control conditions, usual care, etc.).
Outcome	- Changes in physical activity levels, dietary intake, smoking behaviour, alcohol consumption, stress levels, or sleeping behaviour<br>- Changes in physiological outcomes (i.e. weight, lung function, respiratory symptoms, exercise capacity and muscle strength)<br>- Changes in quality of life
Study design	Intervention studies (i.e. single group pre-post studies, non-randomised trial studies, randomised controlled trials).

PubMed search strategy

(Counseling[MeSH] OR "counseling*"[tiab] OR "lifestyle intervention*"[tiab] OR "lifestyle program*"[tiab] OR "lifestyle modification*"[tiab] OR "lifestyle change*"[tiab] OR "behaviour intervention*"[tiab] OR "behavior intervention*"[tiab] OR "behavioural intervention*"[tiab] OR "behavioral intervention*"[tiab] OR "behaviour change intervention*"[tiab] OR "behavior change intervention*"[tiab] OR "multifactorial intervention*"[tiab] OR "multiple risk factor intervention*"[tiab] OR "lifestyle coaching program*"[tiab] OR "health coaching program*"[tiab])

OR

(((Exercise[MeSH] OR exercis*[tiab] OR "physical activit*"[tiab] OR training*[tiab]) **AND** (Diet[MeSH] OR diet*[tiab] OR nutrition*[tiab] OR "food intake"[tiab] OR "nutrient intake"[tiab]))

OR

((Exercise[MeSH] OR exercis*[tiab] OR "physical activit*"[tiab] OR training*[tiab]) **AND** (Smoking[MeSh] OR smoking cessation[MeSH] OR smoking[tiab] OR cigarette*[tiab] OR tobacco[tiab] OR smoke*[tiab]))

OR

((Exercise[MeSH] OR exercis*[tiab] OR "physical activit*"[tiab] OR training*[tiab]) **AND** (Alcohol Drinking[MeSH] OR alcohol[tiab]))

OR

((Exercise[MeSH] OR exercis*[tiab] OR "physical activit*"[tiab] OR training*[tiab]) **AND** (Sleep[MeSH] OR sleep*[tiab]))

OR

((Exercise[MeSH] OR exercis*[tiab] OR "physical activit*"[tiab] OR training*[tiab]) **AND** (Stress, Psychological[MeSH] OR stress*[tiab]))

OR

((Diet[MeSH] OR diet*[tiab] OR nutrition*[tiab] OR "food intake"[tiab] OR "nutrient intake"[tiab]) **AND** (Smoking[MeSh] OR smoking cessation[MeSH] OR smoking[tiab] OR cigarette*[tiab] OR tobacco[tiab] OR smoke*[tiab]))

OR

((Diet[MeSH] OR diet*[tiab] OR nutrition*[tiab] OR "food intake"[tiab] OR "nutrient intake"[tiab]) **AND** (Alcohol Drinking[MeSH] OR alcohol[tiab]))

OR

((Diet[MeSH] OR diet*[tiab] OR nutrition*[tiab] OR "food intake"[tiab] OR "nutrient intake"[tiab]) **AND** (Sleep[MeSH] OR sleep*[tiab]))

OR

((Diet[MeSH] OR diet*[tiab] OR nutrition*[tiab] OR "food intake"[tiab] OR "nutrient intake"[tiab]) **AND** (Stress, Psychological[MeSH] OR stress*[tiab]))

OR

((Smoking[MeSh] OR smoking cessation[MeSH] OR smoking[tiab] OR cigarette*[tiab] OR tobacco[tiab] OR smoke*[tiab]) **AND** (Alcohol Drinking[MeSH] OR alcohol[tiab]))

OR

((Smoking[MeSh] OR smoking cessation[MeSH] OR smoking[tiab] OR cigarette*[tiab] OR tobacco[tiab] OR smoke*[tiab]) **AND** (Sleep[MeSH] OR sleep*[tiab]))

OR

((Smoking[MeSh] OR smoking cessation[MeSH] OR smoking[tiab] OR cigarette*[tiab] OR tobacco[tiab] OR smoke*[tiab]) **AND** (Stress, Psychological[MeSH] OR stress*[tiab]))

OR

((Alcohol Drinking[MeSH] OR alcohol[tiab]) **AND** (Sleep[MeSH] OR sleep*[tiab]))

OR

((Alcohol Drinking[MeSH] OR alcohol[tiab]) **AND** (Stress, Psychological[MeSH] OR stress*[tiab]))

OR

((Sleep[MeSH] OR sleep*[tiab]) **AND** (Stress, Psychological[MeSH] OR stress*[tiab])))

AND (Lung Diseases, Obstructive[MeSH] OR "COPD"[tiab] OR "chronic obstructive airway disease*"[tiab] OR "COAD"[tiab] OR "chronic airflow obstruction*"[tiab] OR emphysema*[tiab] OR "chronic bronchitis"[tiab] OR asthma*[tiab])

AND

((Randomized Controlled Trial[Publication Type] OR Controlled Clinical Trial[Publication Type] OR Clinical Study[Publication Type] OR Clinical Trial[Publication Type] OR Clinical Trial Protocol[Publication Type])

OR

("clinical study"[tiab] OR "clinical trial"[tiab] OR "quantitative study"[tiab] OR "follow up study"[tiab] OR "longitudinal study"[tiab] OR "treatment outcome"[tiab] OR "prospective study"[tiab] OR "qualitative study"[tiab])

OR

((random*[tiab] AND (controlled[tiab] OR control[tiab] OR placebo[tiab] OR versus[tiab] OR vs[tiab] OR group[tiab] OR groups[tiab] OR comparison[tiab] OR compared[tiab] OR arm[tiab] OR arms[tiab] OR crossover[tiab] OR cross-over[tiab]) AND (trial[tiab] OR study[tiab])) OR ((single[tiab] OR double[tiab] OR triple[tiab]) AND (masked[tiab] OR blind*[tiab])))

OR

((("pre post"[tiab] OR "before after"[tiab]) AND (trial[tiab] OR study[tiab] OR design[tiab])) OR ("pre-test post-test"[tiab]))

OR

((non-randomised[tiab] OR non-randomized[tiab]) AND (trial[tiab] OR study[tiab] OR design[tiab]))**)**

Embase search strategy

1. lifestyle modification/
2. (counseling* or "lifestyle intervention*" or "lifestyle program*" or "lifestyle modification*" or "lifestyle change*" or "behaviour intervention*" or "behavior intervention*" or "behavioural intervention*" or "behavioral intervention*" or "behaviour change intervention*" or "behavior change intervention*" or "multifactorial intervention*" or "multiple risk factor intervention*" or "lifestyle coaching program*" or "health coaching program*").ti,ab,kw.
3. 1 OR 2
4. exp physical activity/ or exp exercise/ or exercis*.ti,ab,kw. or "physical activit*".ti,ab,kw. or training*.ti,ab,kw.
5. nutrition/ or exp diet/ or exp dietary intake/ or dietary pattern/ or exp food intake/ or nutritional counseling/ or nutrition*.ti,ab,kw. or diet*.ti,ab,kw. or "food intake".ti,ab,kw. or "nutrient intake".ti,ab,kw.
6. exp smoking/ or smoking.ti,ab,kw. or cigarette*.ti,ab,kw. or tobacco.ti,ab,kw. or smoke*.ti,ab,kw.
7. alcohol consumption/ or drinking behavior/ or alcohol.ti,ab,kw.
8. exp sleep/ or sleep*.ti,ab,kw.
9. exp mental stress/ or stress*.ti,ab,kw.
10. 4 and 5
11. 4 and 6
12. 4 and 7
13. 4 and 8
14. 4 and 9
15. 5 and 6
16. 5 and 7
17. 5 and 8
18. 5 and 9
19. 6 and 7
20. 6 and 8
21. 6 and 9
22. 7 and 8
23. 7 and 9
24. 8 and 9
25. 3 or 10 or 11 or 12 or 13 or 14 or 15 or 16 or 17 or 18 or 19 or 20 or 21 or 22 or 23 or 24
26. exp bronchitis/ or exp lung emphysema/ or exp obstructive lung disease/
27. ("copd" or "chronic obstructive airway disease*" or "coad" or "chronic airflow obstruction*" or emphysema* or "chronic bronchitis" or "asthma*").ti,ab,kw.
28. 26 or 27
29. exp clinical trial/ or "clinical study".ti,ab,kw. or "clinical trial".ti,ab,kw. or "quantitative study".ti,ab,kw. or "follow up study".ti,ab,kw. or "longitudinal study".ti,ab,kw. or "treatment outcome".ti,ab,kw. or "prospective study".ti,ab,kw. or "qualitative study".ti,ab,kw.
30. random*.ti,ab,kw.
31. (controlled or control or placebo or versus or vs or group or groups or comparison or compared or arm or arms or crossover or cross-over).ti,ab,kw.
32. (trial or study).ti,ab,kw.
33. 30 and 31 and 32
34. (single or double or triple).ti,ab,kw.

35. (masked or blind*).ti,ab,kw.
36. 34 and 35
37. 33 or 36
38. ("pre post" or "before after").ti,ab,kw.
39. (trial or study or design).ti,ab,kw.
40. 38 and 39
41. "pre-test post-test".ti,ab,kw.
42. 40 or 41
43. ("non-randomised" or "non-randomized").ti,ab,kw.
44. (trial or study or design).ti,ab,kw.
45. 43 and 44
46. 29 or 37 or 42 or 45
47. 25 and 28 and 46
48. 47 and "Article" [Publication Type]

PsycInfo search strategy

((TI (counseling* OR "lifestyle intervention*" OR "lifestyle program*" OR "lifestyle modification*" OR "lifestyle change*" OR "behaviour intervention*" OR "behavior intervention*" OR "behavioural intervention*" OR "behavioral intervention*" OR "behaviour change intervention*" OR "behavior change intervention*" OR "multifactorial intervention*" OR "multiple risk factor intervention*" OR "lifestyle coaching program*" OR "health coaching program*") **OR** AB (counseling* OR "lifestyle intervention*" OR "lifestyle program*" OR "lifestyle modification*" OR "lifestyle change*" OR "behaviour intervention*" OR "behavior intervention*" OR "behavioural intervention*" OR "behavioral intervention*" OR "behaviour change intervention*" OR "behavior change intervention*" OR "multifactorial intervention*" OR "multiple risk factor intervention*" OR "lifestyle coaching program*" OR "health coaching program*")

OR

(((DE "Physical Activity" OR DE "Actigraphy" OR DE "Exercise" OR DE "Exercise" OR DE "Aerobic Exercise" OR DE "Weightlifting" OR DE "Yoga" OR TI (exercis* OR "physical activit*" OR training*)) **AND** (DE "Nutrition" OR DE "Diets" OR DE "Meat Consumption" OR DE "Vegan Diet" OR DE "Vegetarian Diet" OR DE "Weight Control" OR DE "Eating Behavior" OR TI (diet* OR nutrition* OR "food intake" OR "nutrient intake"))) OR
((DE "Physical Activity" OR DE "Actigraphy" OR DE "Exercise" OR DE "Exercise" OR DE "Aerobic Exercise" OR DE "Weightlifting" OR DE "Yoga" OR AB (exercis* OR "physical activit*" OR training*)) **AND** (DE "Nutrition" OR DE "Diets" OR DE "Meat Consumption" OR DE "Vegan Diet" OR DE "Vegetarian Diet" OR DE "Weight Control" OR DE "Eating Behavior" OR AB (diet* OR nutrition* OR "food intake" OR "nutrient intake"))))

OR

(((DE "Physical Activity" OR DE "Actigraphy" OR DE "Exercise" OR DE "Exercise" OR DE "Aerobic Exercise" OR DE "Weightlifting" OR DE "Yoga" OR TI (exercis* OR "physical activit*" OR training*)) **AND** (DE "Tobacco Smoking" OR DE "Passive Smoking" OR DE "Smoking Cessation" OR TI (smoking OR cigarette* OR tobacco OR smoke*))) OR
((DE "Physical Activity" OR DE "Actigraphy" OR DE "Exercise" OR DE "Exercise" OR DE "Aerobic Exercise" OR DE "Weightlifting" OR DE "Yoga" OR AB (exercis* OR "physical activit*" OR training*)) **AND** (DE "Tobacco Smoking" OR DE "Passive Smoking" OR DE "Smoking Cessation" OR AB (smoking OR cigarette* OR tobacco OR smoke*))))

OR

(((DE "Physical Activity" OR DE "Actigraphy" OR DE "Exercise" OR DE "Exercise" OR DE "Aerobic Exercise" OR DE "Weightlifting" OR DE "Yoga" OR TI (exercis* OR "physical activit*" OR training*)) **AND** (DE "Drinking Behavior" OR DE "Alcohol Drinking Patterns" OR DE "Binge Drinking" OR DE "Social Drinking" OR DE "Underage Drinking" OR TI (alcohol))) OR
((DE "Physical Activity" OR DE "Actigraphy" OR DE "Exercise" OR DE "Exercise" OR DE "Aerobic Exercise" OR DE "Weightlifting" OR DE "Yoga" OR AB (exercis* OR "physical activit*" OR training*)) **AND** (DE "Drinking Behavior" OR DE "Alcohol Drinking Patterns" OR DE "Binge Drinking" OR DE "Social Drinking" OR DE "Underage Drinking" OR AB (alcohol))))

OR

(((DE "Physical Activity" OR DE "Actigraphy" OR DE "Exercise" OR DE "Exercise" OR DE "Aerobic Exercise" OR DE "Weightlifting" OR DE "Yoga" OR TI (exercis* OR "physical activit*" OR training*)) **AND** (DE "Sleep" OR DE "Dreaming" OR DE "Napping" OR DE "NREM Sleep" OR DE "REM Sleep" OR DE "Sleep Onset" OR DE "Sleep Quality" OR DE "Snoring" OR TI (sleep*))) OR
((DE "Physical Activity" OR DE "Actigraphy" OR DE "Exercise" OR DE "Exercise" OR DE "Aerobic Exercise" OR DE "Weightlifting" OR DE "Yoga" OR AB (exercis* OR "physical activit*" OR training*)) **AND** (DE "Sleep" OR DE "Dreaming" OR DE "Napping" OR DE "NREM Sleep" OR DE "REM Sleep" OR DE "Sleep Onset" OR DE "Sleep Quality" OR DE "Snoring" OR AB (sleep*))))

OR

(((DE "Physical Activity" OR DE "Actigraphy" OR DE "Exercise" OR DE "Exercise" OR DE "Aerobic Exercise" OR DE "Weightlifting" OR DE "Yoga" OR TI (exercis* OR "physical activit*" OR training*)) **AND** (DE "Stress" OR DE "Psychological Stress" OR TI (stress*))) OR
((DE "Physical Activity" OR DE "Actigraphy" OR DE "Exercise" OR DE "Exercise" OR DE "Aerobic Exercise" OR DE "Weightlifting" OR DE "Yoga" OR AB (exercis* OR "physical activit*" OR training*)) **AND** (DE "Stress" OR DE "Psychological Stress" OR AB (stress*))))

OR

(((DE "Nutrition" OR DE "Diets" OR DE "Meat Consumption" OR DE "Vegan Diet" OR DE "Vegetarian Diet" OR DE "Weight Control" OR DE "Eating Behavior" OR TI (diet* OR nutrition* OR "food intake" OR "nutrient intake")) **AND** (DE "Tobacco Smoking" OR DE "Passive Smoking" OR DE "Smoking Cessation" OR TI (smoking OR cigarette* OR tobacco OR smoke*))) OR
((DE "Nutrition" OR DE "Diets" OR DE "Meat Consumption" OR DE "Vegan Diet" OR DE "Vegetarian Diet" OR DE "Weight Control" OR DE "Eating Behavior" OR AB (diet* OR nutrition* OR "food intake" OR "nutrient intake")) **AND** (DE "Tobacco Smoking" OR DE "Passive Smoking" OR DE "Smoking Cessation" OR AB (smoking OR cigarette* OR tobacco OR smoke*))))

OR

(((DE "Nutrition" OR DE "Diets" OR DE "Meat Consumption" OR DE "Vegan Diet" OR DE "Vegetarian Diet" OR DE "Weight Control" OR DE "Eating Behavior" OR TI (diet* OR nutrition* OR "food intake" OR "nutrient intake")) **AND** (DE "Drinking Behavior" OR DE "Alcohol Drinking Patterns" OR DE "Binge Drinking" OR DE "Social Drinking" OR DE "Underage Drinking" OR TI (alcohol))) OR
((DE "Nutrition" OR DE "Diets" OR DE "Meat Consumption" OR DE "Vegan Diet" OR DE "Vegetarian Diet" OR DE "Weight Control" OR DE "Eating Behavior" OR AB (diet* OR nutrition* OR "food intake" OR "nutrient intake")) **AND** (DE "Drinking Behavior" OR DE "Alcohol Drinking Patterns" OR DE "Binge Drinking" OR DE "Social Drinking" OR DE "Underage Drinking" OR AB (alcohol))))

OR

(((DE "Nutrition" OR DE "Diets" OR DE "Meat Consumption" OR DE "Vegan Diet" OR DE "Vegetarian Diet" OR DE "Weight Control" OR DE "Eating Behavior" OR TI (diet* OR nutrition* OR "food intake" OR "nutrient intake")) **AND** (DE "Sleep" OR DE "Dreaming" OR DE "Napping" OR DE "NREM Sleep" OR DE "REM Sleep" OR DE "Sleep Onset" OR DE "Sleep Quality" OR DE "Snoring" OR TI (sleep*))) OR
((DE "Nutrition" OR DE "Diets" OR DE "Meat Consumption" OR DE "Vegan Diet" OR DE "Vegetarian Diet" OR DE "Weight Control" OR DE "Eating Behavior" OR AB (diet* OR nutrition* OR "food intake" OR "nutrient intake")) **AND** (DE "Sleep" OR DE "Dreaming" OR DE "Napping" OR DE "NREM Sleep" OR DE "REM Sleep" OR DE "Sleep Onset" OR DE "Sleep Quality" OR DE "Snoring" OR AB (sleep*))))

OR

(((DE "Nutrition" OR DE "Diets" OR DE "Meat Consumption" OR DE "Vegan Diet" OR DE "Vegetarian Diet" OR DE "Weight Control" OR DE "Eating Behavior" OR TI (diet* OR nutrition* OR "food intake" OR "nutrient intake")) **AND** (DE "Stress" OR DE "Psychological Stress" OR TI (stress*))) OR
((DE "Nutrition" OR DE "Diets" OR DE "Meat Consumption" OR DE "Vegan Diet" OR DE "Vegetarian Diet" OR DE "Weight Control" OR DE "Eating Behavior" OR AB (diet* OR nutrition* OR "food intake" OR "nutrient intake")) **AND** (DE "Stress" OR DE "Psychological Stress" OR AB (stress*))))

OR

(((DE "Tobacco Smoking" OR DE "Passive Smoking" OR DE "Smoking Cessation" OR TI (smoking OR cigarette* OR tobacco OR smoke*)) **AND** (DE "Drinking Behavior" OR DE "Alcohol Drinking Patterns" OR DE "Binge Drinking" OR DE "Social Drinking" OR DE "Underage Drinking" OR TI (alcohol))) OR ((DE "Tobacco Smoking" OR DE "Passive Smoking" OR DE "Smoking Cessation" OR AB (smoking OR cigarette* OR tobacco OR smoke*)) **AND** (DE "Drinking Behavior" OR DE "Alcohol Drinking Patterns" OR DE "Binge Drinking" OR DE "Social Drinking" OR DE "Underage Drinking" OR AB (alcohol))))

OR

(((DE "Tobacco Smoking" OR DE "Passive Smoking" OR DE "Smoking Cessation" OR TI (smoking OR cigarette* OR tobacco OR smoke*)) **AND** (DE "Sleep" OR DE "Dreaming" OR DE "Napping" OR DE "NREM Sleep" OR DE "REM Sleep" OR DE "Sleep Onset" OR DE "Sleep Quality" OR DE "Snoring" OR TI (sleep*))) OR ((DE "Tobacco Smoking" OR DE "Passive Smoking" OR DE "Smoking Cessation" OR AB (smoking OR cigarette* OR tobacco OR smoke*)) **AND** (DE "Sleep" OR DE "Dreaming" OR DE "Napping" OR DE "NREM Sleep" OR DE "REM Sleep" OR DE "Sleep Onset" OR DE "Sleep Quality" OR DE "Snoring" OR AB (sleep*))))

OR

(((DE "Tobacco Smoking" OR DE "Passive Smoking" OR DE "Smoking Cessation" OR TI (smoking OR cigarette* OR tobacco OR smoke*)) **AND** (DE "Stress" OR DE "Psychological Stress" OR TI (stress*))) OR ((DE "Tobacco Smoking" OR DE "Passive Smoking" OR DE "Smoking Cessation" OR AB (smoking OR cigarette* OR tobacco OR smoke*)) **AND** (DE "Stress" OR DE "Psychological Stress" OR AB (stress*))))

OR

(((DE "Drinking Behavior" OR DE "Alcohol Drinking Patterns" OR DE "Binge Drinking" OR DE "Social Drinking" OR DE "Underage Drinking" OR TI (alcohol)) **AND** (DE "Sleep" OR DE "Dreaming" OR DE "Napping" OR DE "NREM Sleep" OR DE "REM Sleep" OR DE "Sleep Onset" OR DE "Sleep Quality" OR DE "Snoring" OR TI (sleep*))) OR
((DE "Drinking Behavior" OR DE "Alcohol Drinking Patterns" OR DE "Binge Drinking" OR DE "Social Drinking" OR DE "Underage Drinking" OR AB (alcohol)) **AND** (DE "Sleep" OR DE "Dreaming" OR DE "Napping" OR DE "NREM Sleep" OR DE "REM Sleep" OR DE "Sleep Onset" OR DE "Sleep Quality" OR DE "Snoring" OR AB (sleep*))))

OR

(((DE "Drinking Behavior" OR DE "Alcohol Drinking Patterns" OR DE "Binge Drinking" OR DE "Social Drinking" OR DE "Underage Drinking" OR TI (alcohol)) **AND** (DE "Stress" OR DE "Psychological Stress" OR TI (stress*))) OR
((DE "Drinking Behavior" OR DE "Alcohol Drinking Patterns" OR DE "Binge Drinking" OR DE "Social Drinking" OR DE "Underage Drinking" OR AB (alcohol)) **AND** (DE "Stress" OR DE "Psychological Stress" OR AB (stress*))))

OR

((DE "Sleep" OR DE "Dreaming" OR DE "Napping" OR DE "NREM Sleep" OR DE "REM Sleep" OR DE "Sleep Onset" OR DE "Sleep Quality" OR DE "Snoring" OR TI (sleep*)) **AND** (DE "Stress" OR DE "Psychological Stress" OR TI (stress*))) OR
((DE "Sleep" OR DE "Dreaming" OR DE "Napping" OR DE "NREM Sleep" OR DE "REM Sleep" OR DE "Sleep Onset" OR DE "Sleep Quality" OR DE "Snoring" OR AB (sleep*)) **AND** (DE "Stress" OR DE "Psychological Stress" OR AB (stress*)))))

AND ((DE "Chronic Obstructive Pulmonary Disease" OR DE "Bronchial Disorders" OR DE "Pulmonary Emphysema" OR DE "Asthma") OR **TI** ("COPD" OR "chronic obstructive airway disease*" OR "COAD" OR "chronic airflow obstruction*" OR emphysema* OR "chronic bronchitis" OR asthma*) OR **AB** ("COPD" OR "chronic obstructive airway disease*" OR "COAD" OR "chronic airflow obstruction*" OR emphysema* OR "chronic bronchitis" OR asthma*))

AND

((MR "clinical trial" OR MR "quantitative study" OR MR "followup study" OR MR "longitudinal study" OR MR "treatment outcome" OR MR "prospective study" OR MR "qualitative study")

OR

(DE "Clinical Trials" OR TI ("clinical study" OR "clinical trial" OR "quantitative study" OR "follow up study" OR "longitudinal study" OR "treatment outcome" OR "prospective study" OR "qualitative study") OR AB ("clinical study" OR "clinical trial" OR "quantitative study" OR "follow up study" OR "longitudinal study" OR "treatment outcome" OR "prospective study" OR "qualitative study"))

OR

(((TI (random*) AND TI (controlled OR control OR placebo OR versus OR vs OR group OR groups OR comparison OR compared OR arm OR arms OR crossover OR cross-over) AND TI (trial OR study)) OR (TI (single OR double OR triple) AND TI (masked OR blind*))) **OR**

((AB (random*) AND AB (controlled OR control OR placebo OR versus OR vs OR group OR groups OR comparison OR compared OR arm OR arms OR crossover OR cross-over) AND AB (trial OR study)) OR (AB (single OR double OR triple) AND AB (masked OR blind*))))

OR

(((TI ("pre post" OR "before after") AND TI (trial OR study OR design)) OR TI ("pre-test post-test")) OR
((AB ("pre post" OR "before after") AND AB (trial OR study OR design)) OR AB ("pre-test post-test")))

OR

((TI (non-randomised OR non-randomized) **AND** TI (trial OR study OR design)) OR
(AB (non-randomised OR non-randomized) **AND** AB (trial OR study OR design)))))

Table S2 Descriptive table including details of the included studies and the described combined lifestyle interventions. Studies are reported based on number of lifestyle factors targeted (high to low) and ordered according to study design.

Authors, Year, Reference	Country of implementation CLI	Study design	Patient characteristics						Intervention details	Control group
			Participants, n	Age, mean (sd)	Sex (female), n (%)	BMI, mean (sd)	Current smokers, n (%)	FEV1, mean (sd)		
COPD										

**Kheirabadi et al., 2008**	Iran, Isfahan	Randomised controlled trial	Active: 21<br>Control: 21	56.6 (5.7)<br>56.2 (4.1)	8 (38.1)<br>5 (23.8)	/	/	/	**Duration: 8 weeks**<br>Eight 60-90 minutes educational sessions with 1-week interval in 3-4 member groups. Intervention comprised of<br>- educational sessions on basic information about the disease, side effects and proper use of drugs, respiratory techniques to minimize dyspnoea and use of a self-management program in different conditions.<br>- behaviour modification focused on a healthy lifestyle, smoking cessation, avoiding places with air pollution, healthy sleep, nutritional and sexual habits, stress management, free time activities, traveling, simple regular exercise program at home and behavioural interventions focusing on common issues like independence, decreased self-esteem, feeling insecure, limited relation with family and friends.	Usual care - not further specified

**Walters et al., 2013**	Australia, Tasmania	Randomised controlled trial	Active: 90 Control: 92	68.2 (7.9)	41 (45)	26.3 (4.9)	43 (48)	54.0 (13.4) *% predicted	**Duration: 12 months** The intervention existed of health mentoring (cognitive behavioural basis) and involved five core components: (1) Psychoeducation about common psychological reactions to COPD diagnosis and treatment; (2) self-management skills training, including goal setting, action planning and problem solving skills to manage setbacks; (3) cognitive coping skills training to identify and challenge negative COPD-related cognitions that impede self-management; (4) communication skills to facilitate discussion between the health mentor (HM) and the patient; and (5) promoting self-efficacy to manage chronic illness. Participants set medium-term to long-term goals in collaboration with their health mentor using a specified framework of health behaviour targets, namely: Smoking, Nutrition, Alcohol, Physical activity, Psychosocial well-being and Symptom management (‘SNAPPS’). Individualized ‘action’ plans were set up to reach their goals and there was constant review and revision of the action plan. The predictedetermined schedule for mentor telephone calls to a participant was 16 (total no) ×30 min over 12 months, with increasing time between calls.	Received their usual care as provided by a GP plus regular monthly phone calls from a research nurse. The telephone calls did not provide specific psychological advice or skills training.

**Wilson et al., 2015**	UK, Norfolk	Randomised controlled trial	Active: 73<br>Control: 75	67.3 (15.1)<br>69.3 (8.9)	32 (43.8)<br>25 (33.3)	28.8 (5.7)<br>28.2 (6.0)	/	/	**Duration: 1 year**<br>All patients underwent an **outpatient PR program** for 8 weeks before randomization consisting of weekly 1h of supervised exercise training and 1h educational sessions on relaxation, physiology, medication, emotions, nutrition etc. Patients received recommendation to preform home based exercises. At the end of 8 weeks, patients were randomised to either the maintenance program or to the control group.<br>Maintenance program: patients received one 2h session, conducted every 3 months comprising of 1h education and 1h of exercise training. This was supervised tailored exercise training followed by a home based exercise training prescription.<br>Education sessions- ‘Keeping Well', ‘Keeping Active', 'Keeping Going' covered smoking cessation, healthy eating, the importance of exercise, coping and dealing with psychological issues. Participants also received an invitation to attend Norwich Breathe Easy Group.	8 week outpatient PR program and after randomisation standard care, advise to exercise at home and an invite to attend Norwich Breathe Easy Group.

**Jonsdottir et al., 2015**	Iceland, Reykjavik	Randomised controlled trial	Active: 48<br>Control: 52	59.41 (4.66)<br>58.67 (4.39)	29 (60.4)<br>25 (48.1)	/	24 (50.0)<br>36 (69.2)	54.02 (17.58)<br>60.85 (17.26)<br>*%predicted post bronchodilation	**Duration: 6 months**<br>Partnership-based **self-management programme**, consisted of:<br>- Patient/family conversations: Three to four 30-45 minutes semi-structured conversations with a clinical nurse specialist. Content included: understanding main concerns about the patients' health, symptoms, how it affects daily life, discussing nature of the disease and its management, quitting smoking, and negative feelings and issues associated with the disease, and possibilities for patient/family to prevent further decline of disease.<br>- Smoking cessation treatment: At least one face-to-face conversation with a clinical nurse specialist followed by ≥3 telephonic or face-to face conversations, number and length varied depending on the patient needs. Content: Recommended practice emphasizing psychosocial support combined with pharmacological treatment.<br>- Group meetings: Presentations by research team, provision of written educational material, presentation by a volunteer with COPD, group discussions with 5-15 patient/family members, lasting 2 hours. Content included: quit smoking assistance, knowledge about, skills and motivation to undertake activities to maintain and improve well-being (physical exercise, sleep-rest-activity-relaxation, nutrition, anxiety, depression).	Traditional health care: services provided by general practitioners at primary health care centres and visits to lung physicians based on referral from general practitioners or self-initiated appointments.

**Finnerty et al., 2001**	UK, not further specified	Randomised controlled trial	Active: 36<br>Control: 29	70.4 (8.0)<br>68.4 (10.4)	11 (30.6)<br>10 (34.5)	23.8 (6.5)<br>26.2 (6.2)	2 (5.5)<br>6 (20.7)	41.2 (19.2)<br>41.2 (16.2)<br>*% predicted	**Duration**: **6 weeks**<br>**Outpatient PR** included 2 visits weekly; a 2-h education visit and a 1-h exercise visit. The intervention was conducted by a physiotherapist, occupational therapist, respiratory specialist nurse, and dietitian.<br>- The physiotherapist assisted aerobic activity exercise sessions and gave advice to perform home exercises once or twice daily at least five times a week.<br>- Dietary assessment and advice: progress in any advised change in eating habits was assessed every week.<br>- Consultation with the occupational therapist once weekly for the first four weeks.<br>- Counselling on anxiety management and relaxation techniques in week 2, sleep problems in week 5 and discussion on relaxation techniques in week 6 by a liaison nurse counsellor. Additionally, participants were also invited to attend the local “Breathe Easy” club, which convenes monthly and subsequent “drop-in” exercise sessions at 8 weeks, 9 weeks, and 10 weeks.	Patients reviewed routinely as medical outpatients
**Bendstrup et al., 1997**	Denmark, Esbjerg	Randomised controlled trial	Active: 16<br>Control: 16	64 (3)<br>65 (2)	7 (43.8)<br>7 (43.8)	/	9 (56)<br>7 (44)	1.02 (0.06)<br>1.04 (0.07)<br>*L·min-1	**Duration: 12 weeks**<br>**Out-patient PR:**<br>- Physiotherapist guided exercise training of 1h duration three times a week for 12 weeks. It included strength training (both of upper and lower limbs and back muscle), backwards walking, crossed/uncrossed side-ways walking,	Details not specified

									and running forwards and backwards were used to improve co-ordination and balance, stair climbing used for endurance training and stretching. The physiotherapist individualized the training, according to the needs and resources of each patient. The patients were encouraged to train at home and to continue training after the intervention period.<br>- Educational sessions (total 12 sessions) to optimize disease management, also included sessions by a psychologist, social worker and dietitian (one session each).<br>- Two group classes on occupational therapy.<br>- Smoking cessation intervention: Participants wishing to stop smoking were offered free transdermal nicotine patches, according to established recommendations. Benefits and problems of smoking cessation were taught at the beginning of the programme, with two reinforcement sessions halfway through the programme. A folder with advice was also available. A physician and an occupational therapist were in charge of sessions, the latter offering practical alternatives to smoking.	

**Dheda et al., 2004**	UK, London	Randomised controlled trial	Active: 10<br>Control: 15	68.4 (5.7)<br>71.3 (8.3)	/	/	/	44.7 (21.8)<br>39.0 (11.8)<br>*% predicted	**Duration: 6 months**<br>Regular outpatient follow-up with a respiratory nurse and/or chest physician at least 4 times in a 6 months period (at three, six, eight, 12 or 16 weeks). The following interventions were made at some or all of these visits: spirometry with reversibility, review of inhaler technique and peak flow diary, ambulatory oxygen assessment, smoking cessation advice, steroid trial, nebuliser assessments, review of medication for the addition of long acting B2 agonists and theophyllines, advice about nutrition and exercise, and introduction to a patient support group.	Visited primary care team on "need to" basis
**Theander et al., 2009**	Sweden, not further specified	Randomised controlled trial	Active: 12<br>Control: 14	66 (6)<br>64 (6)	9 (75)<br>4 (28)	24.3 (3.9)<br>25 (3.4)	3 (25)<br>3 (21)	35.1 (7.6)<br>32.3 (9.5)<br>*%predicted post bronchodilat ion	**Duration: 12 weeks**<br>Intervention implemented by a multidisciplinary team, comprising a physiotherapist, dietician, occupational therapist and a nurse. Intervention comprised of:<br>- 2 weekly physiotherapy sessions of approximately 1h long, including both aerobic and strength training. After one month, an individualized home training programme was provided in addition to the physiotherapy sessions.<br>- Individual dietary advice by a dietitian, including education on the importance of adequate intake of energy and nutrients. Patients with a body mass index below 20 received additional nutritional supplementation (respifor 3x125 mL/day containing 635 kJ/100 mL) and a patient with obesity	The control group did not receive any of the multidisciplinary rehabilitation programmes or care from the multidisciplinary professionals who performed the rehabilitation programme.

									(BMI>30) received special nutritional advice. There were a total of three sessions, at weeks 2, 4 and 8. Each session was about 1h long.<br>- Counselling by an occupational therapist. At baseline, the patient and the occupational therapist identified and discussed specific activities involving self-care, productivity and leisure that caused the patients' difficulties. The occupational therapist also educated the patients about energy-saving techniques. There were a total of three sessions, at weeks 2, 4 and 8. Each session was about 1h long.<br>- The nurse educated the patients about their disease, how to manage medications, exacerbations and gave self-care advice. Smoking cessation advice was given for smokers. These sessions were conducted twice, at weeks 2 and 4.	
**van Wetering et al., 2010**	The Netherlands, not further specified	Randomised controlled trial	Total (muscle wasted patients)<br>Active: 16<br>Control:23	64.0 (8.7)	15 (38)	21.7 (4.2)	21 (53.8)	54.7 (15.2) *%predicted	**Duration: 4-month** standardized, supervised **rehabilitation** phase and a 20-month active maintenance phase<br>The **INTERCOM trial** was offered by local dieticians and physiotherapists in the proximity of the patient's home and by respiratory nurses from the hospital. It included<br>- 2 weekly physiotherapist visits during the first 4 months (30 min per visit) for intensive supervised exercise training.<br>- Scheduled counselling (at the start of the intervention and after 1, 2, and 4 months) by a dietician and a standardized nutritional therapy to	The usual care group received pharmacotherapy according to accepted guidelines, a short smoking cessation advice by their chest physician, and if they met the criteria for nutritional

									enhance the efficacy of rehabilitation. Nutritional therapy consisted of 3 oral liquid (3 125 mL) supplements per 24 hours containing 564 kcal in total for a period of 4 months.<br>- Individualized education program and standardized smoking cessation counselling by a respiratory nurse for active smokers.<br><br>During the 20-month maintenance phase:<br>- physiotherapist visit once a month<br>- 4 dietician consultations (after 6, 9, 12, and 24 months), nutritional supplements were continued upon indication.	support, a verbal recommendation to improve dietary intake.

**Zwar et al., 2012**	Australia, Sydney	Randomised controlled trial	Active: 234<br>Control: 217	65.8 (10.3)<br>64.4 (10.3)	124 (53)<br>111 (51.2)	/	74 (31.6)<br>61 (28.1)	/	**Duration: 6 months**<br>Two nurses, specifically recruited and trained for this study, worked in partnership with GPs to implement the intervention. Patients in the intervention group received two home visits and five telephone contacts from the nurse and a minimum of two consultations with their GP over a six-month period. The nurse and GP met face to face on two occasions and further consultation between the nurse and GP took place by telephone monthly or more frequently as needed to discuss progress and problems of the patients involved. Based on the nurse assessment and discussion with the patient of goal setting and action planning, an individualized care plan was made. It contained relevant components of smoking cessation, pulmonary rehabilitation, nutrition, psychosocial issues, patient education, comorbidities and complications of COPD.	Patients received usual care, which was defined as processes normally followed by the GP and the patient regarding review, pharmacologic al therapy and management of COPD.
**Kruis et al., 2014**	The Netherlands (western part)	(Cluster) Randomised controlled trial	Active: 554<br>Control: 532	68.2 (11.3)<br>68.4 (11.1)	274 (49.5)<br>227 (42.7)	/	179 (34.8)<br>196 (38.7)	67.7 (20.3)<br>67.9 (20.5)<br><br>*% predicted	**Duration: 24 months**<br>**RECODE trial**<br>The intervention was delivered at the cluster level. General practitioners, practice nurses, and specialised physiotherapists in the intervention group received a two day training course on incorporating integrated disease management in practice. Each practice team designed a specific time contingent plan in a group discussion	The control group continued usual care (ased on the 2007 national primary care COPD guidelines).

									with their multidisciplinary members. They decided which elements of integrated care they wanted to start implementing first, who would be responsible for which part of the interventions, and which steps to take to integrate integrated disease management into their daily practice. Patients in the intervention group received integrated disease management implemented by the multidisciplinary team consisting of at least three members: the GP, the practice nurse, and physiotherapist. Depending on the team needs, a collaborating pulmonary physician and dietician were added to the intervention team.<br>Essential components of effective COPD-disease management (COPD-DM) included: motivational interviewing, smoking cessation counselling, physical (re)activation and nutritional support. The intensity of the integrated disease management programme for individual patients depended on health status, personal needs, and preferences, as well as on the capacity of the general practice team. As a result, patients with severe disease or at high risk were encouraged to receive multiple interventions, whereas other (for example, stable) patients had only regular control visits.	

**Zwar et al., 2016**	Australia, Sydney	(Cluster) Randomised controlled trial	Active: 144<br>Control: 110	66.5 (10.7)<br>65.4 (11.0)	56 (38.9)<br>46 (41.8)	/	41 (28.5)<br>37 (33.6)	72.1 (17.2)<br>77.8 (16.5)	**Duration: 12 months**<br>All practice nurses, before randomization, received training in case finding for diagnosis of COPD. Nurses and GPs from intervention practices attended further workshops to receive practical education in team-based management of COPD. These additional workshops for GPs and practice nurses in the GP-practice nurse partnership intervention were provided by the study team including a nurse academic, a GP academic, and a respiratory physician. The intervention workshops comprised a full day for practice nurses, a distance learning activity for GPs, and an evening workshop for both practice nurses and GPs. Care planning templates were provided in an electronic form for use by the nurse and GP in computerized clinical record systems. The templates were designed for smokers and non-smokers respectively and had prompts for assessment, goals and actions based on the COPD guidelines. GPs completed a computer-based distance learning activity on the management of COPD according to guidelines. Finally, a 3-hour combined workshop for GPs and PNs focused on each PN-GP team clarifying the individual elements of their roles. Each practice was provided with a copy of national guidelines (COPD-X).<br>In the intervention practices, the practice nurse worked in partnership	After the case-finding training for nurses, staff in control practices received no further intervention other than GPs in these practices being mailed a copy of the COPD-X guidelines.

									with the GP and patient to develop a careplan for patients newly-diagnosed as having COPD. The workbooks provided during the intervention group training workshops prompted the nurse and GP to include relevant components of the following in the care plan:<br>1. Smoking cessation: advice based on Smoking Cessation Guidelines for Australian General Practice<br>2. Immunization (influenza and pneumococcal vaccination)<br>3. Exercise: recommendation about regular exercise for all patients regardless of COPD severity; patients with moderate or severe COPD provided with details of their local pulmonary rehabilitation provider.<br>4. Medication review<br>5. Nutrition: Advice about diet and exercise and referral to a dietician if appropriate for overweight or underweight patients.<br>6. Psychosocial issues: identification and management of anxiety and/or depression.<br>7. Co-morbidities and complications of COPD will be assessed and managed.<br>8. Patient education: patients will be provided with written information about COPD (from Australian Lung Foundationhttp://www.lungfoundation.com.au/) and its management and with information about local patient support groups which provide	

									emotional support and self-management information.	

**Zhang et al., 2020**	China, Zunyi	Randomised controlled trial	Active: 85<br>Control: 89	65.53 (6.64)<br>66.31 (7.91)	20 (23.5)<br>22 (24.7)	/	20 (23.5)<br>20 (22.5)	46.37 (10.69)<br>44.05 (12.71)<br><br>*% predicted	**Duration: 3 months**<br>The intervention consisted of 4 main components:<br>(1) Physical exercise twice a week for 40–55 minutes each time, provided by a physiotherapist, Tai chi mentor, and respiratory nurse:<br>Upper-limb exercise: elastic band–resistance exercise and breath training;<br>Lower-limb exercise: bicycle ergometer, treadmill; and Modified tai chi exercise<br>(2) Smoking cessation: Two sessions (group and individual intervention) provided by a psychologist and respiratory nurse<br>(3) Self-management education every 2 weeks provided by a physician, respiratory nurse, nutritionist on: COPD knowledge, Physical exercise at home, Symptom management, Medication-taking instruction and adherence, Lifestyle change, Nutrition support, and Long-term oxygen therapy.<br>(4) Psychosocial support: Two sessions provided by a psychologist and respiratory nurse including a group activity to facilitate communication among patients, and education on emotional coping strategies.<br><br>After the intensive intervention, long-term follow-up (24 months) was provided by a respiratory nurse. Its rationale was based on hospital-outreach intervention programs that	Usual care: discharge education about self-management, exercise training, medication, and seeking health care when necessary. Each patient in this group got a pamphlet addressing self-management of COPD, including symptom recognition, smoking cessation, physical exercise, medication use, oxygen therapy, and nutrition. Contact information was printed in the pamphlet for a health-

									aim the realization of continuous care among hospital, community, and home. Each patient received an individualized home-exercise prescription. Although home exercise was not supervised by professionals, each patient got an exercise diary to record daily exercise. Periodical telephone follow-up (once every 1–2 weeks) and home visits (once every 1–3 months) were provided. During every telephone or home-visit follow-up, home-exercise adherence, symptom changes, medication adherence, and smoking adherence were recorded by the nurse. Instructions were given to the patient based on the diaries and records, and reminders of necessary outpatient follow-up were given to patients who may suffer deterioration of COPD or need medication prescription. If telephone follow-up showed the patient's exercise or medication adherence was not ideal or his/her health status was not good, home-visiting frequency would be increased.	counselling service.

**Zhu et al., 2021**	China, Jiangsu	Randomised controlled trial	Active: 40<br>Control: 41	72.57 (4.98)<br>72.32 (5.26)<br>72.54 (4.91)	12 (30)<br>14 (34)	/	/	/	**Duration**: **6 months**<br>Community-based **rehabilitation** (CBR) combined with outpatient rehabilitative treatment. The intervention was developed according to the patients' age, physical conditions, and severity of COPD<br>- Rehabilitation education: included lectures to encourage patients to quit smoking and drinking alcohol and to encourage the patients to develop good habits<br>- Exercise: personalized exercise plans comprised of regular walking, cycling, using a treadmill<br>- Psychological counselling: help of psychologists for patients with negative emotions such as emotional anxiety, depression, and boredom	Regular outpatient rehabilitative treatment - not further specified

**Mitchell et al., 2014**	UK, Leicester	Randomised controlled trial	Active: 89<br>Control: 95	69 (8.0)<br>69 (10.1)	35 (39)<br>48 (50.5)	28.05 (5.6)<br>27.09 (4.9)	18 (20)<br>21 (22)	56.04 (16.76)<br>59.60 (17.42)<br><br>*% predicted	**Duration**: **6 weeks**<br>**Self-management program**:<br>- SPACE for COPD manual (to follow independently at home) including educational material ("How to get/stay fit", "Managing your stress", "Healthy eating"). Acquisition of skills was promoted through goal-setting strategies, coping planning and case studies.<br>- Home exercise programme Introduction to the programme was done by a physiotherapist during a 30–45-min consultation. Participants were advised how to use the manual at home and the exercise regime was described by the physiotherapist in detail. Participants received telephone calls from the physiotherapist at 2 and 4 weeks into the programme, with the aim of reinforcing skills and providing encouragement to progress. Goal-setting strategies, and motivational interviewing techniques were used.	Usual care - not further specified

**Benzo et al., 2016**	USA, Minnesota	Randomised controlled trial	Active: 108<br>Control: 107	67.9 (9.8)<br>68.1 (9.2)	62 (57)<br>56(52)	/	/	40.5 (17.1)<br>40.3 (17.2)<br>*% predicted	**Duration: 8 weeks**<br>Same health coaching intervention as in pilot study by Benzo et al., 2013. There was one dedicated coach or interventionist per site. The intervention existed of a total of 8 weekly counselling sessions emphasizing self-management, adopting principles of motivational interviewing. There was one face-to-face intervention session of approximate two hours duration, followed by seven pre-scheduled telephonic sessions. Topics discussed during the first visit included the self-management concepts, goal setting, action planning. The coaches asked about what the patient was already doing to be more comfortable living with chronic lung disease and to be and/or stay healthy in general, and they tried to explore the patient's near-term hopes and goals to work on. The key action plan domains included smoking cessation, coping and stress management and increasing physical activity. The details of the coming telephonic sessions were also discussed during the first visit. Additionally, a copy of the book Living a Healthy Life with Chronic Conditions was given to the patients. The patient was provided with a Stamina In Motion Elliptical Trainer to use daily while sitting (aiming for 20 min/d, a dose of exercise associated with improved outcomes in COPD) and instructed on	Usual care and referral for PR

									how to perform three simple upper extremity exercises (five repetitions) from the book.	
**Gurgun et al., 2013**	Turkey, Izmir	Randomised controlled trial	Active: 15<br>Control: 16	64.0 (10.8)<br>67.8 (6 .6)	2 (13)<br>0 (0)	17.8 (2. 4)<br>19.1 (2. 1)	/	41.9 (10.8)<br>39.3 (9.3)<br>*%predicted post broncholilati on	**Duration**: **8 weeks**<br>**PR + Nutritional intervention**:<br>- PR included education (not further specified) and exercise training. Exercise started with warm-up period followed by cycle ergometer (15 min) and treadmill training (15 min), upper and lower extremity strength training (5–10 min), and breathing and relaxation therapies (15–20 min, each) for total 60–80 min/day twice weekly.<br>- Nutritional intervention: patients received three packages of 250-mL nutritional drink consisting of 83.3% energy from carbohydrates, 30% energy from fat and 16.7% energy from proteins per day, and were also encouraged to continue the consumption of their own meal portions. The patients received dietary advice by the dietitian during visits regarding the content of their meals.	Usual care - not further specified

**Lou et al., 2015**	China, rural areas of Xuzhou City	Randomised controlled trial	Active: 4197<br>Control: 4020	61.6 (13.5)<br>61.4 (13.2)	2191 (52)<br>2096 (52)	23.0 (20.1–24.3)<br>22.9 (20.0–24.4)<br><br>*median (IQR)	1,895 (45.2)<br>1,797 (44.7)	55.1 (82.1–45.3)<br>55.5 (8.8–44.6)<br><br>*%predicted	**Duration**: **4 years**<br>- A health management plan was designed for each subject in the management group.<br>- A lecture every 2 weeks, lasting 40-60 minutes focussing on smoking cessation counselling, encouragement for regular exercise, rehabilitation, and psychological counselling. The health education materials, which contained the content of the 48 total lectures, were distributed to the subjects.<br>- Every 2 weeks, general practitioners conducted at least one face-to-face follow-up visit which took place either at the subject's home or at the health-care centres to assess the subject's compliance with the doctor's orders. The general practitioners filed a monthly report on the condition of each subject for a team of professionals.<br>- Professionals (pulmonologists, psychiatrists, rehabilitation specialists, nutritionists, respiratory nurses) assessed those reports and selected a topic of focus for each subject. The assessments were then returned to the general practitioners. This recording and feedback system was designed for continued improvement of the follow-up procedure.<br><br>The one-hundred thirty-six involved general practitioners, working at 7 different health-care centres, in the management group took 2 days of	Received usual care from health care providers and GPs. Follow up telephonic / face-to-face visits every 2 months. Medical management and referral to respiratory specialist when indicated. Content and frequency not standardized.

									training in health management for the intervention.	

**Suhaj et al., 2016**	India, Manipal	Randomised controlled trial	Active: 130<br>Control: 130	60.6 (7.9)<br>61.1 (8.4)	3.1<br>5.6	/	53.8<br>56.9	44.4 (14.5)<br>41.9 (14.7)<br><br>*FEV1% predicted	**Duration: not specified**<br>Patient education was provided by trained clinical pharmacists as well as patient information leaflets (PILs) were distributed for reinforcing the content delivered through counselling. The counselling sessions (one on one, 15–20 min) and PILs laid emphasis on (i) the importance of medication compliance, (ii) need for smoking cessation, (iii) simple exercise, (iv) proper use of inhaler devices and (v) need for timely follow-up by pulmonary medicine department.<br><br>Each patient was followed up for a period of two years. Patients were further persuaded by monthly telephone calls for ensuring medication adherence and timely follow-ups. During follow-up (every six months), patients in the IG were further trained for proper use of inhaler devices and were motivated for medication adherence.	Standard hospital care

**Markun et al., 2018**	Switzerland, Zurich	Randomised controlled trial	Active: 101<br>Control: 115	68 (63–75)<br>67 (60–73)	41 (40.6)<br>47 (40.9)	25.9 ± 5.99<br>25.6 ± 4.63	56 (55.4)<br>64 (55.7)	70 (55–86)<br>65 (51–76)	**Duration: Not specified**<br>The intervention was delivered to GPs in a half-day workshop. It consisted of knowledge refreshment about Swiss COPD guidelines and the distribution of pocket guides; followed by a discussion with GPs and practice assistants on tailoring their individual pathways of COPD care using case vignettes and role plays. It was proposed that GPs use the COPD care bundle as a checklist to remind and tick-off the individual key elements of COPD care in individual patients in order to increase internal motivation for behaviour change.<br>After 6 months, a 3-h refresher workshop was conducted for the practice teams.<br>Key elements of COPD care included:<br>- Smoking cessation advice<br>- Smoking cessation intervention<br>- Influenza vaccination<br>- Ensuring correct inhalation technique<br>- Appropriate pharmacological treatment<br>- Assessment of physical activity<br>- Advice for physical activity<br>- Patient education class referral<br>- Integration of other healthcare providers<br>- Referral to pulmonary rehabilitation<br>- Exacerbation action plan	No intervention delivered to the “usual care” control group

**Jolly et al., 2018**	England, 71 general practices located at Birmingham and West Midlands South, Greater Manchester, West Midlands North, and Oxfordshire or Gloucestershire	Randomised controlled trial	Active: 289<br>Control: 288	70.7 (8.8)<br>70.2 (7.8)	106 (36.7)<br>105 (36.5)	27.1 (4.4)<br>27.4 (4.9)	75 (26)<br>55 (19)	71.2 (18.9)<br>72.1 (18.7)<br><br>*% predicted post bronchodilation	**Duration: 24 weeks**<br>**Telephone health coaching** by nurses with supporting written documents, a pedometer, and a self-monitoring diary. The intervention included education, monitoring, and assessment in order to increase self-efficacy and followed Social Cognitive Theory. The intervention was developed to support self-management in relation to smoking cessation, physical activity increases, correct inhaler use technique, and medication adherence. The first coaching session was within one week of randomization, lasted 35-60 minutes based on the patient's requirement. Follow up telephonic sessions were at weeks 3, 7, and 11 and lasted 15-20 minute and patients received written supportive materials tailored to individual need. Standard written prompts or information were given at weeks 16 and 24. The nurses were trained by the research team for conducting the telephonic counselling sessions.	Usual care + 13 page standard information leaflet about self management of COPD.

**Thom et al., 2018**	USA, San Francisco	Randomised controlled trial	Active: 100<br>Control: 92	60.7 (8.0)<br>61.9 (7.2)	33 (33)<br>33 (35.9)	/	54 (54.6)<br>45 (52.9)	0.55 (0.19)<br>0.60 (0.20)	**Duration: 9 months**<br>Health coaching by trained coaches to support patient self management. Patients received health coaching for 9 months. Health coaches were expected to complete an initial visit within 2–3 weeks of enrolment; to meet in person with the patient at least three additional times over the course of the study; and to have a phone check-in call at least every 3 weeks, including within 2 weeks after each medical visit (minimum of 13 phone check-ins over 9 months). In-person visits could be at the clinic, at the patient's home, or at a public location that afforded sufficient privacy. Additional contacts were guided by patient needs and preferences. The nine-month health coaching intervention focused on enhancing disease understanding and symptom awareness, improving use of inhalers; making personalized plans to increase physical activity, smoking cessation, or otherwise improve disease management; and facilitating care coordination.<br><br>Health coaches were unlicensed health workers who received approximately 100 hours of training over 3 months and fulfilled the criteria of knowledge and skills required for the same purpose. COPD-specific training was delivered by two pulmonary specialists and covered the physiology of COPD; related comorbidities; Global Initiative	Usual care, included regular physician consultation and any other resources offered by their provider or clinic, i.e., access to COPD educators, respiratory therapists, COPD education classes, pulmonary rehabilitation, smoking cessation classes, and pulmonary specialist referrals by the primary care clinician

									for Chronic Obstructive Lung Disease (GOLD) guidelines; and COPD management, including lifestyle changes.	
**Aboumatar et al., 2019**	USA, Baltimore, Maryland	Randomised controlled trial	Active: 120<br>Control: 120	63.9 (9.6)<br>66.0 (10.0)	72 (60.0)<br>76 (63.3)	28.8(23.1-35.3)<br>27.5(23.7-34.1)<br><br>*median (IQR)	49 (40.8)<br>43 (35.8)	33.3 (16.0)<br>35.8 (14.2)<br><br>*% predicted	**Duration: 3 months**<br>Intervention existed of<br>- Transition support to try to ensure that patients and caregivers were prepared for discharge and understood the post-discharge plan of care.<br>- Individualized COPD self-management support to help patients take medications correctly, recognize exacerbations signs and follow an action plan, practice breathing exercises and energy conservation techniques, maintain an active lifestyle, seek help as needed, and stop smoking.<br>- Facilitated access to community programs and treatment services<br><br>Delivered by COPD nurses (i.e., nurses with special training on supporting patients with COPD using standardized tools). The nurses met with the patient (and caregiver whenever possible) during the hospital stay and for 3 months after discharge; provided self-management support and addressed barriers to care.	Usual transitional care provided at the study site. This included assigning a general transition coach to follow up the patient for 30 days after discharge, focusing on adherence to the discharge plan, and connecting to outpatient care.

**Emery et al., 1998**	USA, not further specified	Randomised controlled trial	Active: 29<br>Control: 25	65.4 (6.4)<br>67.4 (7.1)	15 (51.7)<br>13 (52)	/	/	43 (18)<br>39 (16)<br>*% predicted	**Duration: 10 weeks**<br>**Exercise, education, and stress management (EXESM).** Participants met every day for approx. 4h per day during a 5-week period; sessions included 45 minutes of exercise training daily, weekly 4 h educational session on relevant COPD topics and 1h stress management and psychological support by a clinical psychologist driven by cognitive behavioural therapy. Following the initial intensive 5-week period, EXESM participants then participated in 5 more weeks of a less intense regimen consisting of exercise sessions three times per week for 60-90 min and one hour-long weekly stress management class. Thus, over the course of the 10-week intervention period, EXESM participants were expected to attend 37 exercise sessions, 16 educational sessions, and 10 stress management classes.	Waiting list: were advised not to alter activities significantly

**Blumenthal et al., 2014**	USA, North Carolina & Ohio	Randomised controlled trial	Active: 162<br>Control: 164	65.6 (7.9)<br>66.6 (8.7)	61 (37.7)<br>66 (40)	29.1 (6.4)<br>28.6 (6.6)	30 (19)<br>28 (17)	44.7 (16.7)<br>46.0 (16.8)<br>*%predicted	**Duration: 16 weeks**<br>**Coping Skills Training.** Patients and partners received telephonic counselling on cognitive-behavioural coping by a clinical psychologists weekly for 12 weeks and biweekly for 1 month (total 14 counselling sessions). The components of CST included stress education and coping skills training, promotion of physical activity (individualized exercise prescription), and maintenance and generalization. The duration of each session was 30 minutes.	COPD Education delivered via 12 weekly and 2 biweekly telephone calls from a health educator. Topics relevant to COPD including pulmonary physiology, medication usage, nutrition, and symptom management were discussed. Patients did not receive education on coping strategies.

**Bourne et al., 2022**	UK, Leicester	Randomised controlled trial	Active: 97<br>Control: 96	70.1 (8.2)<br>70.1 (8.2)<br>70.5 (8.4)	88 (45.6)<br>45 (46.4)<br>43 (44.8)	27.5 (5.4)<br>27.1 (5.0)<br>27.7 (5.8)	28 (14.5)<br>13 (13.4)<br>15 (15.6)	1.7 (0.6)<br>1.7 (0.6)<br>1.7 (0.6)	**Duration: 5 months**<br>Participants in the intervention group received a SPACE for COPD manual (same intervention as Mitchell et al.) and attended the SPACE for COPD group-based self-management programme (SMP). The programme was structured around the SPACE for COPD manual, which combines both generic self-management skills and disease-specific tasks. The programme was facilitated by two trained HCPs (e.g. physiotherapist, respiratory specialist nurse, occupational therapist, health psychologist) to groups of up to 10 participants and delivered through six 2-hour sessions, over a 5-month period. Earlier sessions were delivered closer together in time, allowing group cohesion to take place, an important factor in optimising group dynamics. As much as possible, the same facilitators delivered all sessions for each group. Sessions were held at community venues, at times and locations to suit group participants to increase retention and engagement in the intervention.<br>The content of the programme included (all sessions included goal setting):<br>Session 1 (week 1) Introduction to SPACE for COPD (The self-management programme contains a range of educational topics to help an individual with COPD understand their condition better. Some of these topics include:	Participants in the control group continued with any usual check-ups/reviews—no additional care was provided or removed from their current access. If participants were referred to PR in the duration of their time in the study, they were not denied access to the programme. No additional advice, information or recommendations were provided to participants in this group.

									information about medication, breathing control, exercise and nutritional advice)<br>Session 2 (week 2) Introducing exercise and managing shortness of breath - including introduction to the walking programme<br>Session 3 (week 4) Continuing exercise and saving energy - including stenght training<br>Session 4 (week 8) Managing stress and emotions and the COPD action plan<br>Session 5 (week 14) Question and answer<br>Session 6 (week 20) Keeping going from here - including maintaining exercise<br>The exercise component of the manual was completed by participants at home.<br><br>Twelve HCPs attended a 1-day training course and were given an HCP delivery manual to guide the content of the sessions.	
**Monteagudo et al., 2013**	Spain, Barcelona	Non-randomised controlled trial	Active: 400<br>Control: 401	70.2 (9.1)<br>69.6 (8.8)<br>70.7 (9.2)	101 (12.6)<br>65 (16.2)<br>36 (9)	28.5 (4.8)<br>28.4 (4.3)<br>28.5 (5.3)	156 (19.5)<br>84 (20.9)<br>72 (18)	55 (17)<br>54 (18)<br>55 (16)	**Duration: not specified**<br>An integrated programme directed at health professionals to improve quality of life and clinical outcomes of their patients with COPD. Education and motivation workshop designed for the 64 health-care professionals (32 clinicians and 32 nurses) assigned to the intervention group. The 20-h workshop consisted of a structured course that included good clinical practice guidelines on COPD, motivational interviewing, smoking	Health-care professionals in the control group did not participate in the workshop and followed standard clinical care.

									cessation, correct use of inhalers, diet counselling, physical exercise and physiotherapy, and the description of the results obtained from audits and patients' interviews (feedback).	
**Da Silva et al., 2018**	Brazil, Fortaleza	Non-Randomised controlled clinical trial	Active: 38<br>Control: 36	67.7 (6.9)<br>66.1 (7.7)	18 (47.4)<br>12 (33.3)	27.1 (6.3)<br>25 (4.2)	/	46.1 (17.4)<br>42.1 (14)<br><br>*% predicted	**Duration: 12 weeks**<br>Outpatient comprehensive **PR** (3 sessions/week, 60 min per session) provided by a multidisciplinary team including a physiotherapist, chest physician, dietician, occupational therapist, psychologist, and social worker. Physical training included stretching exercises for upper and lower limb, strength and endurance training. Nutritional support, psychological counselling and educational sessions about the pathophysiology of COPD, smoking cessation, acute exacerbations, the role of respiratory medication, and importance of physical activity were given by the psychosocial team.	Patients in a waiting list awaiting admission to PR. Received medical management and were informed about the importance of physical activity; not followed by multidisciplinary team.

**Zakrisson et al., 2011**	Sweden, Multi-center study, multiple public health centre clinics were invited to participate	Non-Randomised controlled trial	Active: 49<br>Control: 54	67 (4)<br>68 (5)	24 (49)<br>22 (51)	28 (6)<br>27 (6)	16 (32.7)<br>23 (42.6)	49 (8)<br>49 (8)<br>*% predicted	**Duration: 6 weeks**<br>**Nurse-led multidisciplinary programme (NMP) of PR** delivered at nine primary health care centers. The intervention consisted of weekly sessions of 2 hours duration for 6 weeks (1h theory and 1h physical activity). Disease management education, including importance of adequate nutrition was provided by the nurses. Anxiety, stress management education and physical activity education was provided by a social worker and physiotherapist respectively (one session each). Patients received individual home exercise prescriptions.	Medical management, no other intervention
**Tania et al., 2017**	Switzerland, Valais	Single group pre-post study	57	66.0 (8.3)	25 (43.9)	/	22 (43.1)	57.5 (18.9)<br><br>*% predicted	**Duration: 12 months**<br>Intervention based on the Chronic Care Model (CCM) and the Canadian programme "Living Well with COPD: A Plan of Action for Life", included:<br>(1) Patient education and self-management: six weekly group sessions on self-management education (90– 120 minutes per session). Educators: a respiratory physiotherapist and/or a nurse specialized in self-management support, accompanied by a pulmonologist and a pharmacist for two specific classes. Topics covered disease education, medication, breathlessness and stress management, prevention and	NA

									management of exacerbations, and lifestyle behaviours including physical activity, smoking cessation, healthy diet, good sleep habits, satisfying sexual life and leisure activities. Educational materials of the Canadian programme, including the action plan, were adapted to the local context, in collaboration with McGill University<br>(2) Scheduled follow-up (every 4 –6 weeks)<br>(3) Multidisciplinary approach<br>(4) Information to and training of healthcare professionals<br>(5) Evidence-based COPD care	
**von Leupoldt et al., 2008**	Germany, Hamburg	Single group pre-post study	210	64 (9)	86 (40.9)	26.5 (5.2)	/	53.9 (17.5)<br><br>*% predicted	**Duration**: **3 weeks**<br>Multidisciplinary outpatient **PR** was performed 6 h/day for 5 days/week consisting of exercise (20 h, endurance training on a stationary cycle ergometer, treadmill, and arm cycle ergometer; strength training for upper, lower, trunk, and respiratory muscles), patient education (11 h), nutrition counselling (5 h), breathing therapy (10 h), relaxation therapy (5 h), psychosocial education (3 h), and smoking cessation support (4 h).	NA
**Yohannes et al., 2021**	Not specified	Single group pre-post study	165	72.2 (8.6)	75 (45)	/	/	47.4 (25.2)<br>*% predicted	**Duration: 8 weeks**<br>**PR** comprising biweekly, 2-h sessions. Each weekly session included 1 h of circuit training comprising strengthening and endurance aerobic exercises and 1h of education. Progressive advancement in exercise intensity was based on patient	NA

									tolerance. Educational sessions were conducted in the form of a group seminar, and topics included nutrition, smoking cessation, coping with chronic disease, anxiety, panic management, and relaxation.	
**Santana et al., 2010**	Brazil, not further specified	Single group pre-post study	Total: 41<br>Analysis split into 2 group:<br>(1) ex-smokers: 18<br>(2) current smokers: 23	64.1 (8.7)<br>63.1 (8.3)	9 (50)<br>10 (43)	25.8 (6.7)<br>22.5 (6.89)	0 (0)<br>23 (100)	42.8 (15.7)<br>45.4 (18.1)	**Duration: 3 months**<br>The **PR** program was carried out in three months, with a frequency of three times per week, lasting 60 minutes each, with a total of 36 sessions. In addition to physical training, monthly educational lectures were delivered to address aspects of the disease, activities of daily living, energy conservation, body awareness and nutrition education. The harmful effects of smoking and its role in the maintenance of symptoms were discussed, but there was no standardized smoking withdrawal program or administration of adjuvant drug treatment.<br>The exercise program consisted of: (i) warm-up followed by 20 minutes of aerobic conditioning on stationary bicycles, with modulated intensity according to individual tolerance; (ii) stretching of the muscles to be exercised during the session; (iii) upper and lower limb resistance training at 50% of the maximum load reached in a previous incremental test and an additional 0.5 kg according to participant tolerance; and (iv) cool-off including stretching of the muscles exercised during the session.	NA

**van Boven et al., 2016**	The Netherlands (recruitment across the whole country)	Single group pre-post study	88	68.8 (7.8)	47.8 %	27.1 (5.1)	23 (26)	/	**Duration: 1 year**<br>Participating pharmacies were part of the Connecting Care collaborative (www.connecting-care.nl); they are pharmacies with well established collaborations with their local primary care team including GPs, physiotherapists, dieticians and primary care nurses. The pharmacists who functioned as project lead for the pharmacies received training about COPD pharmacotherapy optimization (with special attention paid to handling non-adherence management), COPD guidelines and referral criteria. The Medication Monitoring and Optimization (MeMO) COPD intervention program included a patient counselling session followed by a second consultation at 3 months and active monitoring for suboptimal adherence at 6 and 9 months. Initial counselling session content: reinforcement of inhalation instructions, provision of additional information on the medication used, counselling on several aspects of adherence, tailored to a specific type of non-adherence, support for cessation of smoking and recommendations regarding self-management. Pharmacists were advised to always recommend physical activity, with or without referral to the physiotherapist. Pharmacists were advised to refer patients with low (<21) or high (>30) BMIs to a dietician. In specific cases	/

									(e.g. after recommendations to alter dosage or inhaler), a follow-up intervention was undertaken as a supplement to the first intervention. After evaluation of a patient’s medication profile, these interventions could include a second patient counselling session or consultation with the GP.	
**Lewis et al., 2019 (pilot study)**	UK, Islington	Single group pre-post study	42	(n=67) : 62 (11.4)	(n = 78): 46 (59)	(n = 40): 24.6 (4.8)	(n = 63) : 26 (41.3)	(n = 28): 59.8 (23.9)<br><br>*% predicted	**Duration: 4 weeks**<br>The intervention consisted of 4 weekly sessions lasting 2 hours, supervised by a senior physiotherapist and rehabilitation assistant. Other Allied Health Professionals, nursing and medical colleagues contributed to the education component of the programme. Each session consisted of a brief introduction, education component, at least 45 min of exercise intervention and a closing debrief and planning period.<br><br>**Education** components included smoking cessation and signposting other services (healthy eating, smoking cessation).<br>**Exercise component**: warm up, strength and endurance exercise and cool down.<br>Closing and planning period: Setting goals and action plans for the next week.	NA

**Clarke et al., 2016**	South-Africa, Worcester	Single group pre-post study	12 (out of 12, 5 dropped out)	57 (31-71)<br><br>*(min, max)	6 (86)	/	3 (42.9)	/	**Duration**: **12 weeks**<br>Weekly home visit by one or more members of an intervention team consisting of a home-based caregiver, a medical student, a physiotherapy or a human nutrition student to provide education on disease management and lifestyle modification.<br><br>Medical Student: Psychoeducation regarding clinical implications of COPD, warning signs of an exacerbation, and self-care in COPD including importance of correct inhaler technique and lifestyle modification, including smoking cessation.<br><br>Physiotherapy students: Developed tailored exercise programme targeting lower and upper body strength, and endurance. Supervised patients every second week, alternating with home-based caregivers.<br><br>Human nutrition students: Consulted patients at baseline. Provided nutritional information materials for discussion on a weekly basis. Evaluated patients' biometrics for nutritional supplementation.	NA

**Boueri et al., 2001**	USA, Colorado, Denver	Single group pre-post study	37	66 (1.2)<br><br>*(se)	28 (76)	/	/	29.6 (1.8)<br><br>*average (±se) % predicted	**Duration: 3 weeks**<br>**PR**: 12 exercise sessions, which included bicycle ergometer exercise training, upper-extremity training, strength training, and stretching. Patients used an electronically braked bicycle ergometer and started training at 50% of the maximum workload achieved during the prerehabilitation exercise test. The goal of cycle ergometry was to maintain a training duration of 20 min; when patients were able to cycle for 20 min, the work on the cycle was increased. Upper-extremity training was performed by repetitively raising and lowering a dowel from the height of the waist to the height of the shoulders (using an interval-training regimen with repetitive periods of exercise and rest as tolerated by the patient; for example, 2 min of exercise and 1 min of rest). When patients were able to perform the upper-extremity exercise for 10 min, a weight of 0.5 lb was added to each arm. Free weights (dumb-bells, cuff weights) and elastic resistance were used for strength training, for a total duration of up to 30 min as tolerated. Six to 10 upper-body and lower-body strength exercises were used based on demonstrated weakness and fatigue in each individual subject.<br><br>Classes and reading material were used to teach and enhance problem solving.	**/**

									Patients were provided ample opportunity to ask questions and raise concerns related to their lung disease and disability. Subjects were encouraged to attend the following nine group classes: understanding COPD, self-management of COPD, nutrition, stress management, breathing techniques, importance of regular exercise, respiratory medications, oxygen therapy, and sexuality. During individual and group sessions with a social worker, the patient and family addressed psychological aspects of COPD, such as the fear of death, feelings of guilt, depression, anxiety, and relationships with spouse and children.	
**Sahin et al., 2016**	Turkey, not further specified	Single group pre-post study<br><br>*results reported separately for patients with low exacerbation risk (group 1) and those experiencing frequent exacerbations (group 2)	Total: 82<br>Group 1: 52<br>Group 2: 30	60 (56-64)<br>64 (58-69)<br><br>* median (IQR)	5 (9.6)<br>3 (10)	26.5 (22-31)<br>26 (22-29)<br><br>* median (IQR)	/	41 (36-62)<br>31 (23-42)<br><br>* median (IQR)	**Duration: 8 weeks**<br>Outpatient **PR** tailored program twice a week consisting of supervised physical exercise training, theoretical training, nutritional intervention, and psychological counselling if needed. The exercises included breathing exercises, treadmill (min. 15 minutes) and cycle training (min. 15 minutes), peripheral muscle training and stretching exercises. Patients also received information on bronchial hygiene techniques, energy conservation, relaxation techniques for reducing dyspnoea and home exercises. Patients were trained at 60%-90% of maximum heart rate with gradual increase in exercise intensity based on patient progress.	NA

**Helvaci et al., 2019**	Turkey, Ankara	Single group pre-post study	30	66.9 (8.8)	13 (43.3)	/	9 (30.0)	43.0 (20.0-84.0)<br><br>*Median (minimum-maximum) % predicted	**Duration: 8 weeks**<br>**COPD education and counseling programme (COPD-ECP)** based on the recommendations of the Turkish Thorax Society and existing literature. An education booklet was given to patients, which had four chapters, the third chapter of the booklet emphasized a healthy lifestyle including balanced nutrition and adequate sleep-rest and energy conservation techniques. In the fourth chapter, the harmful effects of smoking and the benefits of smoking cessation, and various ways to cope with stress were discussed. The intervention was delivered by the primary investigator (registered nurse) via home visits during the first four weeks and telephonic follow up for next four weeks. During home visits the chapters of the booklet were explained to the patients for about 1 hour followed by 15 minutes of discussion and clearing doubts (total duration 75 minutes/ visit). During the next four weeks, patients were counselled via phone based on their requirement and their questions were answered.	NA

**Gagné et al., 2020**	Canada, Québec	Single group pre-post study	54	68 (8)	23 (43)	26.01 (5.37)	/	/	**Duration: not specified**<br>Respiratory educators participated in a Lecture-Based Continuing Education Activity on Self-Management Support (SMS)<br>They attended a 7-h lecture-based CE activity (delivered by an experienced nurse) on how to deliver COPD-specific SMS. Four months after the CE activity, educators provided SMS to individuals with COPD in their everyday practice.<br>SMS components, based on the PRISMS taxonomy:<br>1. Training for practical self-management activities, e.g., to demonstrate how to teach a patient to use and maintain medication delivery devices;<br>2. Provision of action plans for the management of COPD exacerbations, e.g., to demonstrate how to teach a patient to use an action plan;<br>3. Provision of advice and support around lifestyle, including to support patients concerning smoking cessation, providing nutrition counselling, supporting patients in learning to manage stress, anxiety, breathing, and saving energy.<br>4. Regular clinical reviews, e.g., to perform follow-up visits	NA

**Benzo et al., 2013 (pilot study)**	USA, Minnesota	Single group pre-post study (pilot study)	11	70 (7)	/	/	/	40 (20)<br><br>*% predicted	**Duration: 8 weeks**<br>The intervention included weekly in-person sessions over 8 weeks. Each session included self-management coaching (60 minutes first session, 30 minutes subsequent session) and 60 minutes of exercise training. Self-management education was done along with Motivational Interviewing for fostering engagement in self-management, and encouraging behaviour change. The intervention was implemented by 2 interventionists (1 registered nurse, 1 respiratory therapist). In the first session, participants learn key behaviours for COPD management including incorporating a daily practice, a time carved out in the day to perform simple physical exercises (3 or 4 upper extremity exercises and lower extremity movements that could be just walking or using a portable, inexpensive stationary cycle ergometer provided as part of this study). The following 7 sessions involved self-management action planning in which patients selects a self-management domain (coping with fear and other emotions, quitting smoking, effective breathing, managing fatigue , coping with stress, taking medication, increasing physical activity, relaxation and positive thinking, relationships and communication). Action planning continues by collaboratively setting a	NA

									realistic goal that is completed in the next several days.	

**Kaplan et al., 2004**	USA, not further specified (17 centers)	Single group pre-post study	1.218	67	472 (38.8)	/	/	0.68 (0.22) *L	**Duration**: **6-10 weeks**<br>**PR program** consisting of at least 16 to 20 supervised sessions over 6 to 10 weeks carried out at certified rehabilitation centres close to the participant's home. Components of the pulmonary rehabilitation program included the following: (1) a comprehensive evaluation of medical, psychosocial, and nutritional needs; (2) the setting of goals for education and exercise training; (3) exercise training (i.e., lower extremity, flexibility, strengthening, and upper extremity); (4) education about emphysema, medical treatments; (5) psychosocial counselling; and (6) nutritional counselling.	NA
**Ngaage et al., 2004**	UK, East Yorkshire	Single group pre-post study	14	63 (53-74)<br><br>*(min-max)	9 (64.3)	/	/	/<br>*end-stage COPD and were being considered for lung volume reduction surgery	**Duration: 6 weeks**<br>Comprehensive **PR** program including<br>- Education and dietary advice: they were given basic lessons on chest clearance, positioning for breathlessness, breathing control, the use and action of bronchodilators and other medications, and the need for exercise. Each patient underwent a dietary assessment, and a recommendation of caloric, fluid, and supplement intake was made based on normal daily activities, exercises, and medication supervised tailored exercise training twice weekly and education session consisting of disease education and dietary advice.<br>- Exercise program: exercises were supervised by the physiotherapist in a	NA

									group of 3-4 patients twice weekly. The program consisted of a circuit of 10 exercises aimed at the upper- and lower-body muscle groups, postural exercises, and general aerobic exercises. The home exercise program consisted of the exercises that the patient had performed and tolerated well at the hospital sessions. They performed these exercises 2 to 3 times daily on the days they did not attend the hospital. An increasing gradation of exercises was given to each patient to perform at home based on their progress.	
**McDonald et al., 2016 & McLoughlin et al., 2017 (secondary analysis of McDonald et al., 2016)**	Australia, Newcastle	Single group pre-post study	28	67.6 (6.3)	11 (39)	36.1 (4.5)	0 (0) *all ex-smokers with a smoking history of >10 pack years and ceased smoking >6 months prior to study	61.6 (17.1) *% predicted post bronchodilation	**Duration**: **3 months** **Combined diet and exercise intervention**: Hypo calorie diet prescription 3850–5000kJ/day, or up to 5900kJ/day for patients with a BMI>40kg/m2. Two daily meal replacement supplements for BMI up to 40kg/m2, participants with BMI> 40kg/m2 were prescribed three meal replacements accounting for extra calorie intake. Meal replacement provided approx. 870kJ/serve: constituting 45% carbohydrate, 40% protein and 20% fat. Patients consumed the third balanced meal constituting 1200–1750kJ and a further 900–1200kJ throughout the day to meet daily requirement. Recommended protein was 1.2-1.5g/ kg/ adjusted body weight. Patients also received dietitian's counselling through out the intervention. Home based	NA

									strength training sessions were prescribed and supervised by a physiotherapist 3 days a week with one rest day between sessions. Education and counselling was also given by the physiotherapist to optimize the exercise intervention. Patients were advised to maintain an exercise log.	
**Korkmaz et al., 2020**	Turkey, Konya	Single group pre-post study	66	63.27 (7.43)	2 (3.03)	/	/	/	**Duration: 8 weeks**<br>2h **PR** session, 3 days a week consisting of exercise and nutritional support.<br>- 100 to 120 min of intense and comprehensive exercise individualised according to patient need included a combination of endurance and strength exercises. The program was designed to target 70% to 80% of the maximum capacity measured during baseline evaluation.<br>- An expert dietician instructed patients about the importance of nutritional support. To prevent weight loss and restore muscle atrophy, a body composition analysis was initially performed for each patient, and a personalized diet was determined by the expert dietician. In addition to the personalized diet, patients with an FFMI<16 kg/m2 for men and<15 kg/m2 for women received oral nutritional therapy (as 125ml liquid package) three times a day; each serving containing 300 kcal, 18 g of protein (E24%), 11.75 g of fat(E35%) and 30.5 g of carbohydrate(E41%).	NA

**Pagano et al., 2023**	Australia, Syndey	Single group pre-post study	31	75 (9.3)	19 (61)	27.7 (5.3)	5 (16)	75 (18.6)<br><br>*% predicted post BD	**Duration: 3 months**<br>All physiotherapists in the study completed an advanced training workshop in the management of COPD.<br>A brief intervention occurred at three time points and was coordinated by the physiotherapist at each site in collaboration with general practice staff.<br>All participants received the following intervention;<br>- referral to PR if they met the requirement according to the COPD-X guidelines (national guidelines)<br>- physical activity advice and counselling using the 5 A's approach (Ask, Advise, Assess, Assist, Arrange follow-up) according to the Australian Physical Activity and Sedentary Behaviour Guidelines<br>- provision of a pedometer and pedometer diary to monitor PA goals and guide exercise prescription at follow-up appointments with participants instructed to wear the pedometer every day during awake hours and record daily steps in the diary;<br>- individualised smoking cessation advice support and appropriate referral to their GP if required;<br>- review of inhaler technique if applicable; and<br>- provision of education booklets regarding PA guidelines, smoking cessation and general COPD management.	NA

									The physiotherapist also worked in partnership with the GP and patient to develop or review a COPD-specific GP Management Plan (GPMP) and/or a COPD action plan. Participants then returned for a follow-up visit with the physiotherapist at one month to review PA levels and establish a PA goal to progress towards. A final assessment was completed at three months to review progress.	
**Ansari et al., 2020**	Australia, Sydney	Single group pre-post study	50	69.22 (8.43)	25 (50)	28.99 (6.19)	12 (24)	/	**Duration: 6 months**<br>**APCOM study**: self-management education programme for COPD in the context of multi-morbidity. Participating practice nurses (PNs) attended 1-day workshops facilitated by the authors and were trained to deliver the self-management education programme. The PNs were trained to conduct a patient assessment using a template based on the Health Belief Model. This assessment identified the patients' health priorities and was used by the PNs to facilitate development of tailored strategies for the patients to support self-management of COPD in the context of multi-morbidity. During the first PN–patient session (visit 2), individual patient needs were assessed and the intervention tailored accordingly. In the following two sessions (visits 3 and 4), PNs used motivational interviewing to address barriers faced by patients in managing their COPD in the face of co-	NA

									morbidities, and work towards optimising health behaviour. Intervention delivery based on 5As: Ask, Advise, Assess, Assist and Arrange for their patients' health behaviour. Health information and referrals to healthcare providers were provided as necessary. Following the last session, the PNs followed up with the patients via a monthly phone call for 5 months.<br><br>The assessment and planning template for the PN-patient sessions incorporates the HBM's constructs with strategies for self-management of COPD and co-morbidities. The template included cues to<br>1. COPD knowledge<br>2. Relationship between COPD and patients' co-morbidities<br>3. Management and prioritisation of their multiple chronic health conditions<br>4. Managing flare-ups using a COPD Action Plan<br>5. Recommended exercises and pulmonary rehabilitation<br>6. Proper use of inhalers<br>7. Importance of flu and pneumonia vaccination<br>8. Medication review and adherence<br>9. Advice on overall health and wellbeing<br>10. Effects of smoking and benefits of quitting, assistance with quitting and abstinence	

Asthma										
**Vempati et al., 2009**	India, Delhi	Randomised controlled trial	Active: 29<br>Control: 28	33.5 (11.4)<br>33.4 (11.5)	16 (55)<br>8 (29)	23.4 (4.3)<br>22.6 (4.0)	/	70.2 (17.4)<br>62.5 (19.2)<br><br>*% predicted	**Duration: 8 weeks** (2 weeks guided, then follow-up at home)<br>**Yoga-based lifestyle modification and stress management program:** 2 weeks supervised yoga based lifestyle modification training followed by closely monitored home practices for 6 weeks. Initial 2 weeks 4h sessions included practicing yoga, and pranayama for 1h supervised by a qualified yoga instructor, refreshment and building group support for 30 min; lecture and discussion for 2h and meditation for 30 min. Education sessions included stress management, nutrition and relevant health education delivered by physicians with special interest in yoga and mind-body medicine. Participants also received printed material and audio cassettes to supplement live instruction. They were asked to maintain a diary on adherence to yoga practice, dietary advice and rescue medication use, which was reviewed daily.	Received conventional care and were offered a session on health education relevant to their illness

**Ma et al., 2015**	USA, Northern California	Randomised controlled trial	Active: 165<br>Control: 165	47.6 (12.4)<br>47.5 (12.6)<br>47.7 (12.1)	233 (70.6)<br>116 (70.3)<br>117 (70.9)	37.5 (5.9)<br>37.4 (6.0)<br>37.6 (5.7)	5.8<br>7.3<br>4.3<br>*%<br>(n=229)	2.6 (0.8)<br>2.6 (0.8)<br>2.6 (0.8)<br>*L	**Duration: 12 months**<br>The BEWELL intervention dually targeted modest weight loss and increased physical activity and had three successive stages: Intensive (13 weekly in-person group sessions over 4 months), Transitional (two monthly in-person individual sessions), and Extended (three bimonthly or more frequent phone consultations depending on participant needs, preferences, and availability). The intervention was theory-based and goal-oriented. Staff monitored and responded to participants' individual weight-loss needs, preferences, and personal circumstances by counselling them on healthy eating with moderate calorie reductions (by 500–1,000 kcal/d, but daily total calories no less than 1,200 kcal), moderate-intensity physical activity (e.g., brisk walking), and behavioural self-management skills.	Usual care enhanced with a pedometer, a weight scale, and a list of routinely offered Kaiser Permanente in Northern California (KPNC) weight management services, and a KPNC standard asthma self-management educational DVD. The research team made no other attempts to intervene with control participants.

**Toennesen et al., 2018**<br><br>**Bentzon et al., 2019 (Follow-up study of Toennesen et al., 2018)**	Denmark, not further specified	Randomised controlled trial	Total: 125<br>Active: 29<br>Control: 34<br><br>*there were 2 more intervention groups therefore active and control do not add up to total<br><br>Total: 25<br>Active: 15<br>Control: 10	43.7 (13.9)<br>38.2 (12.7)<br><br>47.9 (10.5)<br>41.0 (12.8)	22 (75.9)<br>26 (76.5)<br><br>11 (73.3)<br>3 (30)	26.1 (2.5)<br>25.5 (2.4)<br><br>25.7 (2.6)<br>25.0 (1.9)	1 (3)<br>2 (6)<br><br>0 (0)<br>0 (0)	82.6 (15.2)<br>81.9 (12.3)<br><br>*% predicted<br><br>84.1 (13.4)<br>89.4 (16.0)<br><br>*% predicted	**Duration: 8 weeks**<br>**Exercise + diet intervention**:<br><br>- Exercise intervention: high-intensity interval training using the"10-20-30"concept on indoor spinning bikes 3 times a week, supervised by a qualified sports instructor.<br>- Diet intervention: 5 group counselling sessions (2-6 patients/group) and 1 individual counselling session with a trained study dietician. Diet advised- high protein (25%-28% of energy), low GI (<= 55), anti-inflammatory, i.e., higher amounts of vegetables, fruits, nuts, lean meat, fish, and seafood in accordance with the Shivappa et al. anti-inflammatory index.	Patients in the control group received no intervention and were encouraged to maintain usual physical activity levels and diet.
**Al-Sharif et al., 2020**	Saudi Arabia, Jeddah	Randomised controlled trial	Active: 36<br>Control: 36	39.6 (7.3)<br>38.3 (6.8)	/	31.72 (2.78)<br>31.51 (3.23)	/	1.65 (0.86)<br>1.83 (0.79)<br>*L	**Duration: 12 weeks**<br>45 minutes of treadmill based aerobic exercise training (including 5 minutes warm up and 10 minutes cool down), 3 sessions/ week for 12 weeks. Dietitian supervised diet regime providing 1200Kcal/ day.	No diet and exercise intervention

**Pokladnikova et al., 2013 (pilot study)**	The Czech Republic, not further specified	Randomised controlled trial	Active: 15<br>Control: 12	50.7 (15)<br>54.7 (14.2)	14 (93)<br>11 (92)	26 (4)<br>29 (5)	0 (0)<br>0 (0)	/	**Duration: 8 weeks**<br>The **Self-management program** involved four group meetings on lifestyle changes based on yoga (1.5 hours per session) and four individual sessions on psychotherapy based on Eastern philosophy and ethicotherapy (1 hour per session).<br><br>Lifestyle changes included an asthma-specific diet (Mediterranean style and allergen-free diet), yogic postures (asanas), stress management training encompassing relaxation (shavasana), breathing techniques (pranayamas), meditation, emotion management, and effective communication skills training. Patients were told to practice lifestyle changes every day. Each patient received a workbook and a diary for keeping notes on the program assignments and practices.<br><br>All group sessions were educational and were followed by a discussion and skill training. Yoga and psychotherapy sessions were conducted by a certified yoga instructor and a psychotherapist specialized in spirituality-based cognitive-behavioural therapy.	Standard care

**Tousman et al., 2011**	USA, Virginia	Randomised controlled trial	Active: 21<br>Control: 24	51.4 (14.7)<br>55.0 (10.0)	17 (80.9)<br>14 (58.3)	/	/	/	**Duration: 7 weeks**<br>**Asthma self-management program**<br>7 weekly meetings consisted of interactive discussions, problem-solving, social support and a behaviour modification procedure. Each of the seven 2h meetings were divided into 2 specific components: individual status report (60 min) and discussion topic (60 min).<br><br>Participants also received a self-management behaviour homework assignment to be practiced on a regular basis prior to the next session, which included both asthma-specific goals (based on NHLBI asthma guidelines - trigger avoidance/removal, reading about asthma, peak flow monitoring, and controller medication adherence) and general lifestyle goals. General lifestyle behaviors included 1. Practice 20 min of relaxation, 2. Washing hands with warm water for at least 20 seconds at least 7 times during the day, 3. Exercising for 20 min, and 4. Drinking 64 ounces of water. Participants were asked to mark down points each day for these behaviours. The following week during the ISR, the participants shared their results and received feedback from the group. The program was delivered by facilitators including a psychologist, certified asthma educator working as a clinical nurse specialist, occupational therapist, and physician assistant.	Not specified

**Rasulnia et al., 2017**	USA, Durham, North Carolina	Single group pre-post study	40	50 (21, 78)<br><br>*median (minimum, maximum),	30 (75)	/	6 (15)	/	**Duration: 12 weeks**<br>- Printed material and tools: information booklet on healthy eating principles, interactive booklet encouraging participants to contemplate their goals, external facilitators, and external barriers, booklets to track diet, and physical activity, pedometer, magnet reinforcing healthy eating principles.<br>- Curriculum of easy-to-read, written materials included the topic exercise in week 6, healthy living in week 7, eating right in week 8 and stop smoking in week 10.<br>- Weekly engagement with health advisor/coach to support the received information, using motivational interviewing techniques.	NA

**Johnson et al., 2022**	USA, Vermont and Arizona	Single group pre-post study	43	48 (41-59)<br><br>*median (IQR)	38 (88)	40.3 (34.7-46.8)<br><br>*median (IQR)	/	80 (69-97)<br><br>*median (IQR)% predicted **pre-BD**	**Duration: 6 months**<br>**An online weight loss intervention** included<br>- Individualized calorie and fat intake goals and increased physical activity introduced through weekly group meetings in a synchronous chat (i.e., in real time) led by a registered dietitian.<br>- Weekly lessons and individualized nutrition education emphasized consuming a diet high in fruits, vegetables, and whole grains and low in fat, sugar, salt, and alcohol.<br>- Daily food intake, physical activity, and weight in MyFitnessPal, was recorded by participants, which was monitored by the weight loss facilitator who provided weekly feedback to reinforce or shape new behaviours. Participants were provided with incremental goals to reach the recommended weekly minimum of 200 minutes of moderate intensity exercise by week 9 of the program. Brisk walking was recommended because this form of exercise requires no equipment and can be safely done by most participants.	NA

**Mammen et al., 2022**	USA, New York	Single group pre-post study	30	18–44 year	19 (63.3)	34.36 ± 11.29	11 (36.7)	/	**Duration: 6 months** A multi-component program was designed to support predominantly remote primary care management of asthma. It incorporated 3 technological components to augment usual care including 1. smartphone asthma symptom monitoring, 2.smartphone-based telemedicine follow-up and self-management training (SMT) with a nurse via Zoom video-conferencing, and 3.guideline-based clinical decision support(CDS) software that calculates asthma severity, control, and recommend step-wise therapy based on Expert Panel Report-3 guide-lines. The home telemedicine follow-ups and SMTs were conducted by a nurse with the patient every 2-6 weeks until the asthma was well controlled and follow-up occurred every 2-3 months after asthma control was achieved. Modules on smoking cessation and exercise for managing asthma were included as part of SMT module.	NA

BMI: body mass index, COPD: chronic obstructive pulmonary disease, FEV1: forced expiratory volume in 1 second, FFMI: fat free mass index, GP: general practitioner, PNs: practice nurses, PR: pulmonary rehabilitation

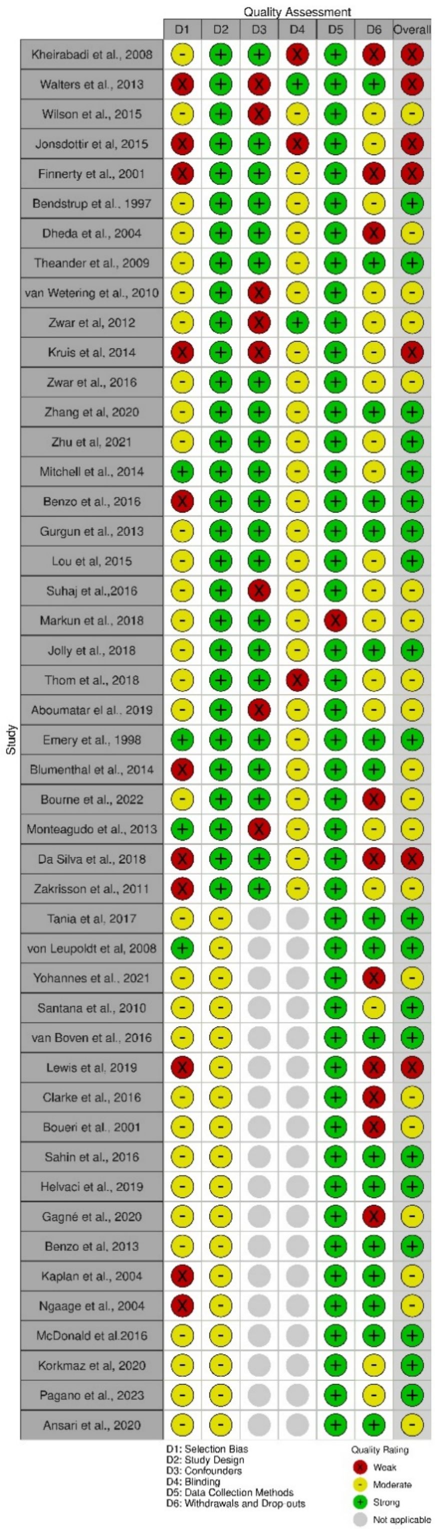

Figure S1 Quality plot for COPD studies
Studies are reported based on number of lifestyle factors targeted (high to low) and ordered according to study design
COPD: chronic obstructive pulmonary disease

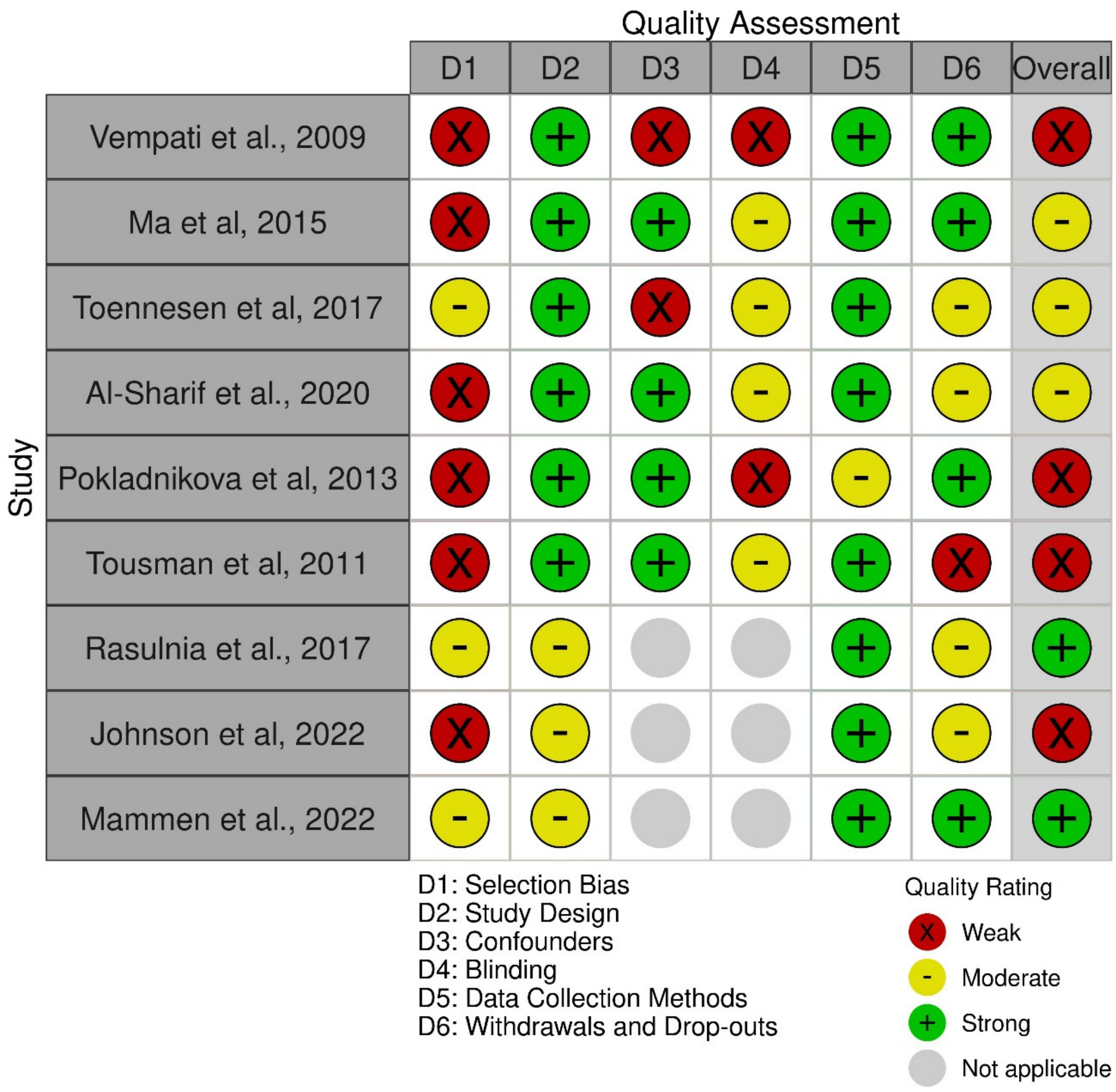

Figure S2 Quality plot for asthma studies
Studies are reported based on number of lifestyle factors targeted (high to low) and ordered according to study design
COPD: chronic obstructive pulmonary disease

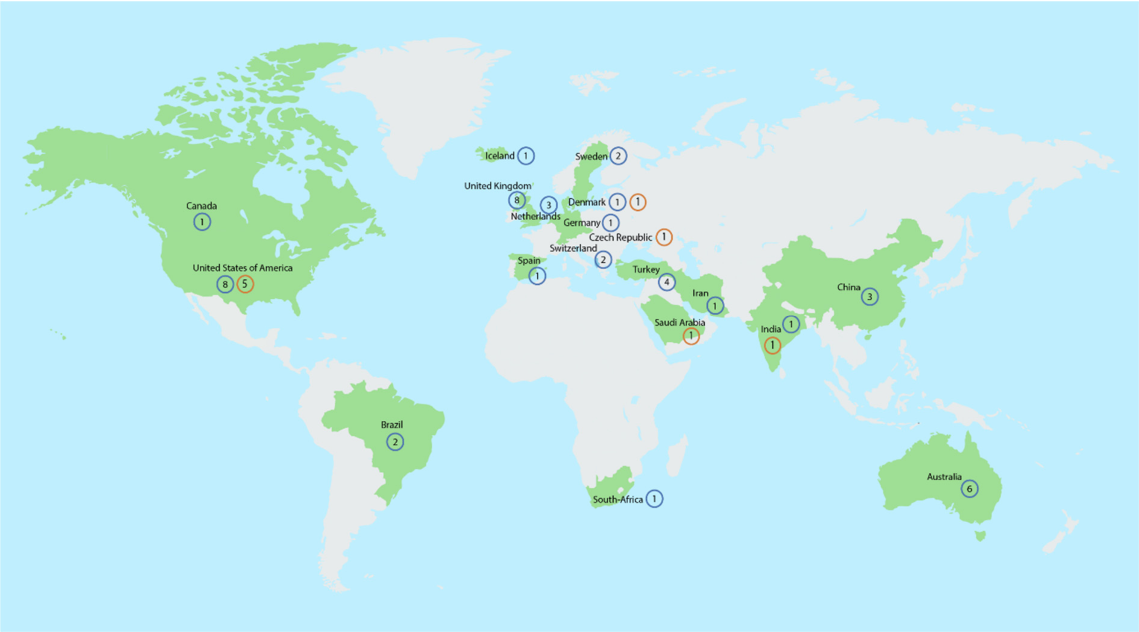

Figure S3 Locations of CLI implementation for asthma and COPD management
Orange: Number of CLIs for asthma management
Blue: Number of CLIs for COPD management

1. Ouzzani M, Hammady H, Fedorowicz Z, et al. Rayyan-a web and mobile app for systematic reviews. *Syst Rev*. 2016;5(1):210. https://doi.org/10.1186/s13643-016-0384-4.

2. McMaster University. Effective Public Health Practice Project (EPHPP). https://merst.healthsci.mcmaster.ca/ephpp/. Date last updated: 2023. Date last accessed: August 19 2022.
